# Heterotypic seeding of Tau fibrillization by pre-aggregated Abeta provides potent seeds for prion-like seeding and propagation of Tau-pathology in vivo

**DOI:** 10.1007/s00401-015-1525-x

**Published:** 2016-01-06

**Authors:** Bruno Vasconcelos, Ilie-Cosmin Stancu, Arjan Buist, Matthew Bird, Peng Wang, Alexandre Vanoosthuyse, Kristof Van Kolen, An Verheyen, Pascal Kienlen-Campard, Jean-Noël Octave, Peter Baatsen, Diederik Moechars, Ilse Dewachter

**Affiliations:** Alzheimer Dementia Group, Institute of Neuroscience, Catholic University of Louvain, 1200 Brussels, Belgium; Department of Neuroscience, Janssen Research and Development, A Division of Janssen Pharmaceutica NV, 2340 Beerse, Belgium; VIB11 vzw Center for the Biology of Disease, KU Leuven, 3000 Leuven, Belgium

**Keywords:** Amyloid beta, Tau, Heterotypic seeding, Prion-like, Alzheimer’s disease

## Abstract

Genetic, clinical, histopathological and biomarker data strongly support Beta-amyloid (Aβ) induced spreading of Tau-pathology beyond entorhinal cortex (EC), as a crucial process in conversion from preclinical cognitively normal to Alzheimer‘s Disease (AD), while the underlying mechanism remains unclear. In vivo preclinical models have reproducibly recapitulated Aβ-induced Tau-pathology. Tau pathology was thereby also induced by aggregated Aβ, in functionally connected brain areas, reminiscent of a prion-like seeding process. In this work we demonstrate, that pre-aggregated Aβ can directly induce Tau fibrillization by cross-seeding, in a cell-free assay, comparable to that demonstrated before for alpha-synuclein and Tau. We furthermore demonstrate, in a well-characterized cellular Tau-aggregation assay that Aβ-seeds cross-seeded Tau-pathology and strongly catalyzed pre-existing Tau-aggregation, reminiscent of the pathogenetic process in AD. Finally, we demonstrate that heterotypic seeded Tau by pre-aggregated Aβ provides efficient seeds for induction and propagation of Tau-pathology in vivo. Prion-like, heterotypic seeding of Tau fibrillization by Aβ, providing potent seeds for propagating Tau pathology in vivo, as demonstrated here, provides a compelling molecular mechanism for Aβ-induced propagation of Tau-pathology, beyond regions with pre-existing Tau-pathology (entorhinal cortex/locus coeruleus). Cross-seeding along functional connections could thereby resolve the initial spatial dissociation between amyloid- and Tau-pathology, and preferential propagation of Tau-pathology in regions with pre-existing ‘silent’ Tau-pathology, by conversion of a ‘silent’ Tau pathology to a ‘spreading’ Tau-pathology, observed in AD.

**Electronic supplementary material:**

The online version of this article (doi:10.1007/s00401-015-1525-x) contains supplementary material, which is available to authorized users.

## Introduction

Brains of AD patients are characterized by the presence of neurofibrillary tangles and amyloid plaques composed of aggregated Tau and Aβ peptides, respectively [46, 47]. In AD, spreading of Tau-aggregation occurs in a very characteristic pattern (Braak stages) along functionally connected brain areas [3], strongly correlating with symptom progression, explaining its use as diagnostic criteria. Combined genetic, biomarker and histopathological data indicate increasing Aβ peptide concentrations as driving force for the conversion from preclinical to mild cognitive impairment (MCI) and finally AD [19, 25, 26, 53], strongly correlating with spreading of Tau-pathology beyond EC and the limbic system to the neocortex, by a poorly understood molecular mechanism. Noteworthy, a spatial dissociation between predominant amyloid- and Tau-pathology exists in (the initial stages of) AD. Amyloid-pathology accumulates predominantly in isocortex (Thal stages) [54], while Tau-pathology (Braak stages) [3] spreads from EC in a characteristic pattern along functionally connected regions.

While mechanistically unclear, Aβ-induced Tau-pathology has been demonstrated reproducibly in in vitro and in vivo models [2, 5, 13, 29, 36, 40, 49, 50]. Indeed, in a human neural stem cell derived 3D culture system, expression of EOFAD mutations induced aggregated Aβ and Tau filaments, with Tauopathy downstream of Aβ formation [5]. Furthermore, different milestone papers have demonstrated amyloid-induced Tau-pathology in in vivo animal models, including following (1) crossing or co-expressing of mutant APP/PS1 with mutant Tau in transgenic mice [2, 29, 36, 40, 49] following (2) injection of pre-aggregated Aβ containing extracts derived from AD patients or APP/PS1 transgenic mice [2], or (3) following injection of synthetic pre-aggregated Aβ [13]. Intriguingly, Aβ-induced Tau-pathology, following injection of pre-aggregated Aβ, induced not only Tau-pathology at the site of injection but also in brain regions remote from, but functionally connected to the injection site [2, 13].

These findings are not only reminiscent of the spatial dissociation between amyloid and Tau-pathology in the initial stages in AD patients, but also reminiscent of the prion-like spreading of Tau-pathology following Tau-seeding, along functional connections [1, 6, 22, 51]. Prion-like properties have been demonstrated for different proteins associated with neurodegenerative disorders [6, 8, 12, 16, 17, 22, 27, 28, 30, 33, 38, 39, 51, 56], including Tau. Prion-like seeding and propagation of Tau-pathology have been demonstrated in vitro and in vivo in different models with synthetic or patient-derived pre-aggregated Tau [6, 16, 17, 22, 28, 39, 44, 51]. Prion-like “seeding” using pre-aggregated Tau fibrils propagates Tau-aggregation by overcoming the rate-limiting nucleation step of Tau-aggregation. Once initiated Tau-pathology propagates rapidly in a prion-like way in in vivo models, and according to a pattern observed in AD, following initiation in EC Tau-pathology spreads to limbic system and cortex [7, 22, 27, 51]. Tau-pathology is thereby propagated to remote but functionally connected brain regions, affecting intrinsic functional neuronal networks, reminiscent of the networks affected in AD and related Tauopathies [1, 22, 51]. Although mechanistically not yet fully resolved, in terms of uptake and spreading of “seeds”, this process has been reproducibly recapitulated in different models. The strong similarity in induction of Tau-pathology by prion-like Tau-seeds and pre-aggregated forms of Aβ in in vivo models (i.e. in crosses of transgenic mice, following injection of pre-aggregated Aβ containing brain extracts from mice and AD patients, and pre-aggregated synthetic Aβ) has incited us to analyze whether pre-aggregated Aβ could directly cross-seed filamentous Tau-aggregation, leading to propagation of Tau pathology. Interestingly, cross-seeding between Tau and alpha-synuclein has been demonstrated previously in vitro and in vivo [11, 15]. Hence, in this work we have analyzed the potential of Aβ-seeds to induce Tau-aggregation and the potential of these Aβ-induced Tau-seeds to seed propagating Tau-pathology in vivo, as a potential mechanism underlying the propagation of Tau-pathology beyond EC in AD.

## Materials and methods

### In vitro generation of monomeric and pre-aggregated seeds

*In vitro generation of pre-aggregated synthetic Aβ 1–42 peptides* Aβ 1–42 peptides were purchased from Bachem (Bachem AG, Switzerland). Aβ monomers and Aβ aggregates (fibrils) were prepared as previously described [52]. Briefly, lyophilized monomeric Aβ 1–42 was resuspended in HFIP (Sigma-Aldrich) and subsequently evaporated for 1 h in a SpeedVac (Thermo Fisher Scientific, Waltham, MA, USA) before its storage in single use aliquots at −20 °C. For Aβ 1–42 aggregation, the stored monomeric Aβ 1–42 was resuspended in DMSO (Sigma-Aldrich) at 5 mM, before dilution to 100 µM in 10 mM HCl solution and incubation 24 h at 37 °C. The aggregation nature of the preparations was assessed by thioflavin T (ThioT) assay, immunoblotting and immuno-EM. For all the experiments only freshly prepared sonicated fibrils (8 pulses of 30 % amplitude) were used.

*In vitro generation of monomeric and pre-aggregated Tau-“Tau seeds”* Tau seeds were generated as previously described [16, 22, 51]. The human truncated 4R Tau, encompassing the 4-repeat microtubule binding domain of Tau with the P301L mutation and a myc tag (K18-P301L; Q244-E372) was generated in *Escherichia coli*, further referred to as Tau^Mono^. Pre-aggregated synthetic Tau, “Tau seeds”, were obtained by incubation of Tau^Mono^ (66.7 µM) with low molecular weight heparin [MP Biomedicals, Santa Ana, CA, USA; (ratio 1:2)] in 100 mM ammonium acetate buffer (pH 7) at 37 °C for 5 days. Before use, fibrillization mixture was centrifuged (100,000*g* for 1 h at 4 °C) and the resultant pellet resuspended in the same buffer without heparin to a final concentration of 333 µM and stored at −80 °C. Successful Tau fibrillization was confirmed by ThioT (Sigma-Aldrich, St. Louis, MO, USA) assay, immunoblotting and immuno-EM. For all experiments Tau seeds were sonicated (8 pulses of 30 % amplitude) before use.

*In vitro generation of pre-aggregated synthetic amylin peptides* Amylin peptides were purchased from Bachem (Bachem AG, Switzerland). Amylin aggregates were prepared as described [42, 43]. Briefly, amylin peptides were dissolved in DMSO to a final concentration of 20 mM and stored at −20 °C. For amylin aggregation, the stored monomeric peptides were dissolved in 25 µM KCl to a final concentration of 20 µM and incubated at 37 °C for 65 h. The aggregation nature of the preparations was assessed by ThioT assay and immunoblotting. For all the experiments only freshly prepared sonicated fibrils (8 pulses of 30 % amplitude) were used.

### Cell culture and Tau aggregation assay

Human kidney-derived QBI-293 (QBiogene, Carlsbad, CA, USA) were grown in Dulbecco’s modified Eagle’s medium (DMEM) supplemented with 10 % (v/v) heat inactivated FBS, 1 % Pyruvate (10 mM), 1 % Penicillin–Streptomycin (PenStrep) and l-glutamine (20 mM). Cells were maintained at 37 °C in humidified atmosphere containing 5 % CO_2_. One day prior to transfection, 80 % confluent cells were trypsinized and then seeded in 10 cm^2^ dishes at 1.5 × 10^6^ cells per well. The growth medium was renewed directly before transfection. DNA mixture containing 2.5 µg pcDNA6-TR and 2.5 µg 2N4R-TauP301L-GFP-pcDNA4/TO was diluted in 500 µL OptiMEM and 15 μL FuGENE^®^ 6 transfection reagent diluted in 500 µL OptiMEM was added. The mixture was incubated for 15 min at room temperature (RT), then added to the cells. After incubation for 24 h, the growth medium was removed and replaced with a new one containing 5 µg/mL blasticidin and 200 µg/mL Zeozin. The cells were cultured until selection was complete. Monoclonal lines were generated by limited dilution. Cells were then grown in full media (DMEM, 10 % FBS; Invitrogen, Life Technologies, Carlsbad, CA, USA) supplemented with penicillin/streptomycin, l-glutamine and pyruvate. For the Tau aggregation assays, cells were plated at 100,000 per well in 12-well plates in medium containing doxycycline (Westburg, Leusden, Netherlands). On the day of aggregates transfection, Tau-seeds and Aβ-seeds were diluted in the respective buffers to a 10 µM solution and sonicated with 8 pulses/30 % amplitude; 80 µL of this solution was added to single use BioPORTER tubes (AMS Biotechnology, Milton, UK), vortexed at low speed for 5 s and incubated 10 min at RT. During the seeds-BioPORTER complexes formation, cells were washed with OptiMEM (Invitrogen) and placed in 250 µL of OptiMEM. 420 µL of OptiMEM was added to the BioPORTER tubes, and 250 µL of the resultant solution was added to the cells. The cells were incubated with the seeds for 4 h, and subsequently 500 µL medium was added (complete medium containing 20 % FBS) and further incubated for 3 days. For experiments using 24-well plates, the volumes were adjusted accordingly. For the experiments using Aβ seeds as catalyst of pre-existing Tau aggregation, two “BioPORTER transfections” were performed. On the first day of the experiment, Tau-seeds were transfected to the cells followed by the transfection of Aβ-seeds 24 h later. For the control condition BioPORTER without Aβ-seeds was added. The cells were further incubated for 2 days.

### Biochemical analysis

For total cell extracts, cells were washed and scraped in OptiMEM and centrifuged at 1000*g* for 5 min at 4 °C. Cell pellets were then resuspended in Triton lysis buffer (1 % TX-100, 50 mM Tris, 150 mM NaCl, pH 7.6) containing proteases and phosphatases inhibitors (F. Hoffman-La Roche AG, Basel, CH) before storage at −20 °C. For the separation of soluble and insoluble Tau, the Triton/SDS method was used as previously described [16, 22, 51]. Briefly, cells were washed with PBS, scraped into Triton lysis buffer containing protease and phosphatase inhibitors and incubated on ice for 15 min. Cell extracts were sonicated and centrifuged at 100,000*g* for 30 min at 4 °C. The supernatants yielded the Triton-soluble fraction (soluble proteins), while the pellets were resuspended, sonicated and vortexed in SDS lysis buffer (1 % SDS, 50 mM Tris, 150 mM NaCl, pH 7.6) and stored as Triton-insoluble fraction, after centrifugation at 100,000*g* for 30 min at 28 °C. For all samples proteins were quantified using BCA Protein Assay kit (Thermo Fisher Scientific, Waltham, MA, USA). Samples were run in precast 4–12 % Bis–Tris gels (MOPS running; Invitrogen) and immunoblotting was performed with anti-Tau P-S202/T205 (AT8 1:500; Thermo Fisher Scientific), anti-total Tau antibody (HT7 1:500; Thermo Fisher Scientific) and anti-actin antibody (1:500; Sigma-Aldrich) and developed using ECL kit (PerkinElmer, Waltham, MA, USA).

### Cytological and immunocytological analysis

Cells were fixed under stringent conditions for the removal of soluble Tau proteins using fixation/extraction buffer (4 % PFA, 4 % sucrose, 1 % TX-100 in PBS) for 10 min. After washing with PBS, cells were blocked (5 % FCS or milk in 0.1 % TX-100 PBS) for 30 min. The specific anti-Tau P-S202/T205 (AT8 1:100) and anti-conformational specific Tau (MC1 1:100, Peter Davies) were incubated for 2 h at RT or overnight at 4 °C followed by staining with Alexa Fluor 568 goat anti-Mouse IgG1 (1:500) or Alexa Fluor 647 goat anti-Mouse IgG1 (1:500) for 1 h at RT. DAPI staining to label nuclei was performed in a PBS washing step. The GFP bleaching experiments were performed as previously described with minor modifications [32]. Briefly, a 5 µM acid-denatured GFP solution (pH 1.5) was prepared by mixing equal amounts of solution A (50 mM Tris–HCl pH 7.5) with solution B (125 mM HCl) and incubated with cells 1 min at RT. Image acquisition was performed using a digital inverted fluorescence microscope (EVOS-xl auto microscope). The 10×, 20× and 40× coverslip corrected lens were used in this study.

### In vitro Tau aggregation assay (cell-free assay)

To assess the ability of Aβ seeds to directly cross-seed Tau aggregation, in vitro Tau fibrillization experiments were performed. Tau^Mono^ (66.7 µM) was incubated with heparin (133 µM), pre-aggregated synthetic Tau seeds (6.67 µM; buffer A), Aβ seeds (6.67 µM; buffer B), amylin seeds (6.67 µM), albumin (6.67 µM), or with buffers alone (diluted as used in the respective seeding conditions). The fibrillization reaction was performed for 3 days at 37 °C with constant agitation at 150 rpm. Aliquots of all reactions were collected at different time points and successful fibrillization of Tau was verified by ThioT assay, sedimentation test and immunogold electron microscopy.

### ThioT assay

The assay was performed as previously described [10, 55]. Briefly, samples were incubated with ThioT solution (5 µM ThioT in 100 mM ammonium acetate buffer pH 7.0) in 96-well plates at RT. Fluorescence was measured with a PerkinElmer Victor X3 (PerkinElmer) with an excitation filter of 440 nm and an emission filter of 486 nm.

### Sedimentation analysis

The sedimentation analysis was performed as previously described with minor modifications [11]. Briefly, samples were ultracentrifuged (100,000*g* for 1 h at 4 °C) and the resultant pellet was resuspended in a volume of buffer corresponding to the volume of supernatant. Western Blot analysis was performed using an anti-total Tau antibody (1:2000; DAKO, Glostrup, Denmark).

### Immunogold electron microscopy

Immunogold labeling was performed as previously described [51]. Pure protein samples of the in vitro Tau fibrillization experiments were applied on 200-mesh carbon-coated grids in drops of 2 µL for 5 min, blotted on filter paper and air-dried before immunogold labeling. Grids were blocked in PBS 0.1 % cold water fish gelatin (PBS-CWFG) for 5 min before incubation with PBS-CWFG diluted primary antibody (Polyclonal Total Human Tau 1:50) for 90 min at RT. After washing five times (2 min) in PBS-CWFG, grids were incubated for 30 min with protein A secondary antibody labeled with 10 nm gold particles (Aurion, Wageningen, Netherlands) diluted 1:50 in PBS-CWFG. Following washes in PBS and ddH2O, grids were negatively stained with 2 % uranyl acetate for 45 s. Grids were examined with a JEOL JEM1400 transmission electron microscopy equipped with an Olympus SIS Quemesa 11Mpxl camera, and images were taken at magnifications of 10×, 20× and 30× k. For co-staining, grids were sequentially incubated for 90 min with the primary mouse anti-Aβ antibody (WO2 1:50) followed by rabbit anti-total human Tau antibody (DAKO Tau 1:50). Subsequently the grids were incubated with secondary antibodies at RT in PBS-CWFG. The secondary antibodies used were, respectively, 10 nm gold coupled goat anti-mouse antibody (1:50 in PBS-CWFG) and 5 nm gold coupled goat anti-rabbit antibody (1:50 in PBS-CWFG). The grids were negatively stained with 2 % uranyl acetate for 45 s and were examined as previously described.

### In vivo seeding experiments

*Animals* TauP301S mice (PS19) [58] expressing the Tau isoform with one N-terminal insert and four microtubule binding repeats (1N4R) with the P301S clinical mutation, driven by the prion protein promotor, were backcrossed with C57B6 mice and used in this study. The TauP301S mice used in our lab develop similar neuropathology to previously reported studies, at around 11 months [22, 50, 58]. Animals were housed on a 12 h light/dark cycle in specific pathogen-free (SPF) facilities with access to food and water ad libitum. Stereotactic injections were performed in 4 months old mice and age-matched littermates were used for analysis at 3 months post-injection. All experiments were performed in compliance with protocols approved by the UCLouvain Ethical Committee for Animal Welfare.

*Stereotactic injections* For stereotactic surgery, 4 months old mice were deeply anesthetized with a mixture of ketamine/xylazine (Ketalar/Rompun). Unilateral (right hemisphere) stereotactic injections were performed in the hippocampal region (A/P, −2.0 mm; L, +1.4 mm; D/V, −1.4 mm) and frontal cortex (A/P, +2.0 mm; L, +1.4 mm; D/V, −1.0 mm); all coordinates are expressed relative to bregma [37]. Sonicated pre-aggregated homotypic Tau-induced Tau-aggregates (66.7 µM), Aβ-induced Tau-aggregates (66.7 µM), non-aggregated or monomeric Tau controls (66.7 µM), Aβ seeds (333 µM) and buffer controls were injected using a 10 µL Hamilton syringe at a speed of 1 μL per min. After injection, the needle was kept in place for additional 5 min before gentle withdrawal.

*Immunohistological analysis* Immunohistological analysis was performed as previously described [41, 50]. Animals were transcardial perfused with ice cold PBS (2 min) by transcardial flushing and brains were dissected and fixed in 4 % PFA in PBS for 24 h at 4 °C. 40 μm thick sagittal sections were cut in a vibrating HM650V microtome (Thermo Fisher Scientific, Waltham, MA, USA). Immunohistochemistry (IHC) was performed on these sections with anti-Tau P-S202/T205 (AT8), anti-Tau P-S212/T214 (AT100) and anti-conformational Tau (MC1, Peter Davies) primary antibodies followed by appropriate Alexa-coupled secondary antibody, as previously described [50]. Staining with Thioflavin S (ThioS; Sigma-Aldrich, St. Louis, MO, USA) and Gallyas silver (all chemicals from Sigma-Aldrich, St. Louis, MO, USA) staining were performed as previously described [50, 51] and were used to demonstrate the “amyloid” nature of Tau aggregates in brain sections. Image acquisition was performed using a digital inverted fluorescence microscope (EVOS-xl auto microscope), using the 4×, 10× and 20× objectives. Image analysis was performed using Image J (National Institutes of Health). Heat maps were generated using the HeatMap Histogram plugin for Image J, as previously described [51]. Briefly, the overview images of AT8 staining of a well-identified section of different mice (*n* = 6 per group) were grouped. Stacked images representing averaged intensities were generated using the Image J stacking tool with the average intensities outcome option. Finally a Gaussian Blur filter of 5.0 was applied.

### Statistical analysis

Statistical analysis was performed using GraphPad Prism 5.0 (GraphPad Software, San Diego, CA, USA). Normality testing was performed for all the data using D’Agostino and Pearson omnibus normality test when possible. Kolmogorov–Smirnov tests were used for smaller sample sizes. According to the results of normality tests appropriate statistical tests were used, including one-way analysis of variance (ANOVA) followed by Tukey’s post hoc test, two-way ANOVA followed by Bonferroni post hoc test and Kruskal–Wallis test followed by Dunn’s multiple comparison post hoc test. The tests used are indicated in the legends of the figures. Data were expressed as mean ± SEM and differences were considered significant when *p* values <0.05.

## Results

### Pre-aggregated Aβ-seeds induce Tau-aggregation in a well-characterized cellular Tau aggregation assay

**Fig. 1:**
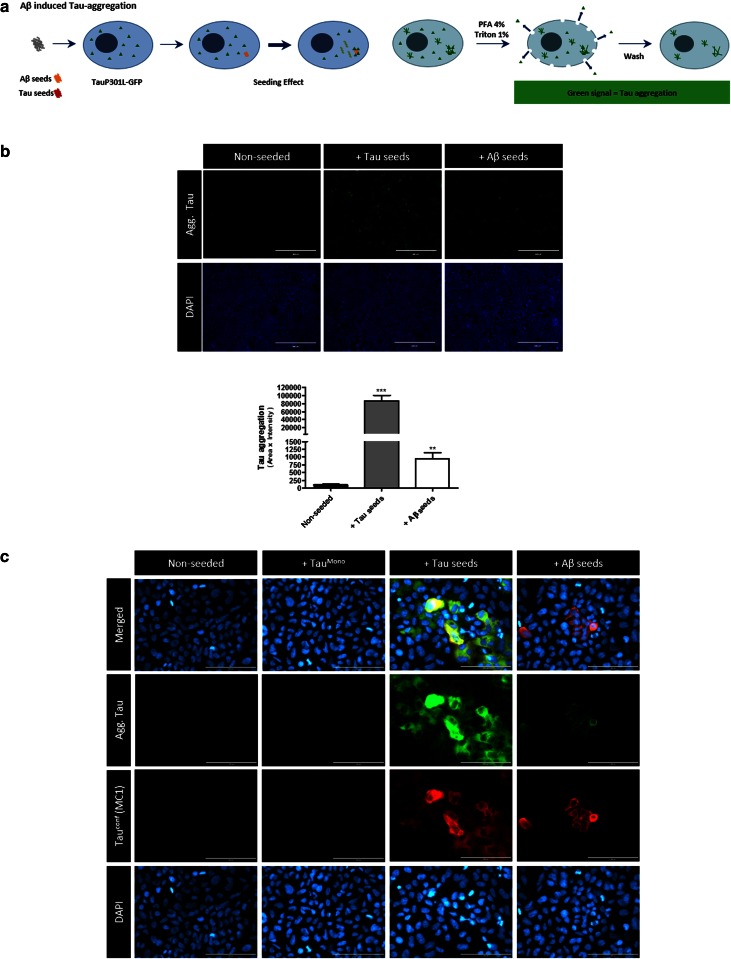
Pre-aggregated Aβ-seeds induce Tau-aggregation in a well-characterized in vitro Tau-aggregation assay. **a** Schematic design of seed induced Tau-aggregation in QBI-293 cells expressing TauP301L-GFP. Pre-aggregated Tau-seeds or Aβ-seeds are added to the cells thereby bypassing the slow nucleation-dependent lag phase. Following stringent extraction using 1 % TX-100 during PFA fixation, soluble forms of Tau are eliminated. The remaining GFP signal represents aggregated GFP-tagged TauP301L. **b** Similar to Tau-seeds, Aβ-seeds induce Tau-aggregation in the in vitro Tau-aggregation assay, albeit less efficiently than Tau-seeds (*scale bar* 400 µm). Quantitative analysis reveals significant Aβ-seed induced Tau aggregation (*n* ≥ 30 fields per condition; Kruskal–Wallis test followed by Dunn’s multiple comparison post hoc test, indicating significant difference compared to non-seeded condition; mean ± SEM are presented; **p value <0.01, ***p value <0.001). **c** Higher magnification images of the different conditions are presented. Aggregated Tau is stained with MC1 antibody, detecting conformationally altered Tau, further confirming the seed induced Tau pathology, absent in non-seeded cells, and not significantly induced in cells treated with monomeric Aβ forms or monomeric Tau forms (Fig. S2) (*scale bar* 100 µm)

To start exploring molecular mechanisms of Aβ-induced Tau-aggregation we used a previously described and characterized cellular Tau-aggregation assay [16, 44, 51]. In this assay, the nucleation-dependent long initial lag phase for Tau-fibrillization in cells is by-passed by the addition of preformed pre-aggregated synthetic Tau-seeds, which accelerate fibrillization of monomeric Tau. Hereto, QBI-293 cells were transfected with the longest human Tau isoform (2N4R) with the P301L mutation, to optimize Tau aggregation, and transduced with preformed fibrils generated from Myc-tagged truncated Tau P301L containing only the four MT-binding repeats—further referred to as Tau-seeds when aggregated and Tau^Mono^ in their monomeric form—using BioPORTER protein delivery reagent. GFP-tagged Tau displayed similar aggregation as non-tagged Tau, and was therefore used in the assay to facilitate detection of Tau-aggregation. Cells transduced with Tau-seeds displayed accumulation of Triton-insoluble phospho-Tau aggregates as demonstrated by Western blotting analysis, while no Tau-aggregates were detected following transduction with BioPORTER only (non-seeded) (Fig. S1). For cytological analysis, a stringent extraction was performed during fixation (using 1 % TX-100) to eliminate soluble Tau forms, leaving aggregated Tau detectable following Tau-seeding with Tau-seeds. These aggregates were also detected by anti-phospho-Tau primary antibody AT8 (S202/T205) and conformation-dependent anti-Tau antibody MC1 (Fig. S1). This assay is well characterized, has been extended to different cell types, is reproducible in different labs and has previously been used to identify the existence of different Tau prion-strains [16, 22, 44, 51]. It was also used to evaluate the potential of cross-seeding of Tau and alpha-synuclein in cells [15, 44, 57]. Here, we used this assay to analyze whether pre-aggregated Aβ seeds could facilitate Tau-aggregation, in a similar way as pre-aggregated Tau-seeds. We used this Tau-aggregation assay for analysis of seeding with pre-aggregated synthetic Tau-seeds, pre-aggregated synthetic Aβ (Aβ 1–42) fragments or BioPORTER alone (Fig. 1). Seeding with pre-aggregated synthetic Tau fragments significantly and robustly induced Tau-aggregation as demonstrated by the presence of Tau-GFP signal following stringent extraction with 1 % TX-100, validating the assay. Strikingly, seeding with pre-aggregated Aβ, further referred to as “Aβ-seeds”, induced Tau-aggregation in a reproducible consistent way (Fig. 1). In contrast to pre-aggregated Aβ-seeds and pre-aggregated Tau-seeds, their monomeric forms (monomeric Tau and monomeric Aβ) did not induce significant Tau-aggregation in this assay (Fig. S2). Notably, Tau-aggregation as a consequence of pre-aggregated Aβ cross-seeding was markedly less strong than Tau-aggregation induced with pre-aggregated Tau-fragments, but was consistently induced in each well in each experiment. This was further confirmed by quantitative analysis demonstrating significantly increased induction of Tau-pathology by Aβ-seeds (Fig. 1).

### Pre-aggregated Aβ-seeds accelerate incipient Tau-aggregation in a cellular Tau aggregation assay

**Fig. 2:**
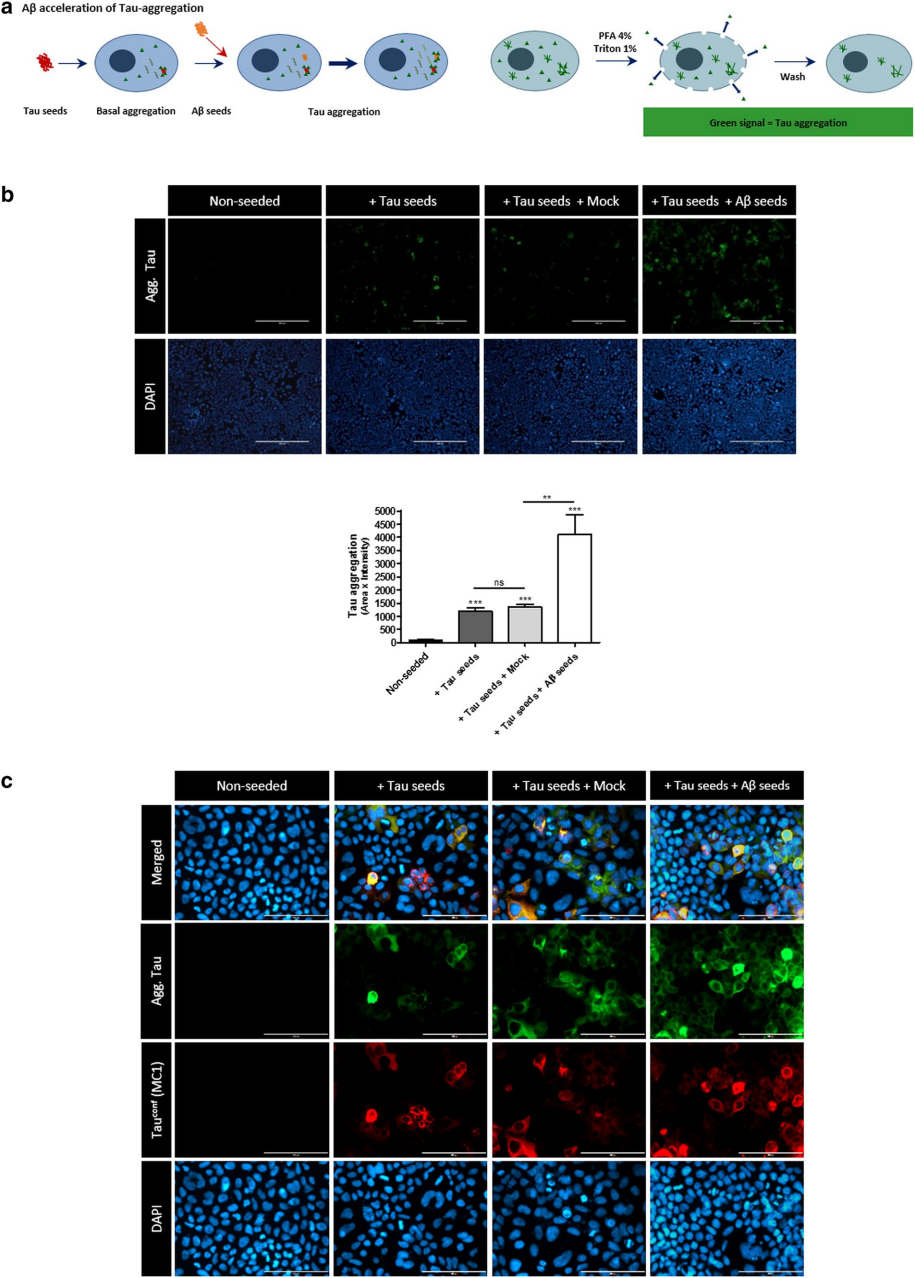
Pre-aggregated Aβ-seeds strongly accelerate incipient or pre-existing Tau-aggregation. **a** Schematic presentation of the assay. Following induction of Tau-aggregation with Tau-seeds in TauP301L-GFP expressing cells, cells are subsequently seeded with Aβ-seeds or control conditions. Analysis is performed by cytological analysis following stringent extraction to eliminate soluble forms of Tau. **b** Seeding with Tau-seeds induced Tau-aggregation as demonstrated previously, however, subsequent seeding with Aβ-seeds strongly catalyzes Tau-aggregation compared to control conditions (“mock”) (*scale bar* 400 µm). Quantitative analysis of Tau-aggregation demonstrates significantly increased Tau-aggregation following subsequent seeding with Aβ-seeds (*n* ≥ 36 fields per condition; Kruskal–Wallis test followed by Dunn’s multiple comparison post hoc test, indicating significant difference in all conditions compared to non-seeded condition, and significant difference between sequential seeding with Aβ and single Tau seeding; mean ± SEM are presented; **p value <0.01, ***p value <0.001). **c** Higher magnifications of the different experimental conditions are provided. Aggregated Tau is further confirmed to display conformational alterations by staining with MC1 antibody (*scale bar* 100 µm)

In view of the fact that Tau-aggregation can be present in locus coeruleus (LC) and entorhinal cortex (EC) already early in life, but only spreads from EC to functionally connected brain regions during conversion to MCI and AD—generally hypothesized due to increasing Aβ burden-, we analyzed whether pre-aggregated Aβ could accelerate propagation of incipient Tau-pathology [3, 19, 31]. Hence, we analyzed whether Aβ-seeds could aggravate incipient Tau-pathology in this cellular Tau-aggregation assay (Fig. 2). Hereto, Tau-seed induced Tau aggregation was followed by an additional seeding with Aβ-seeds. As demonstrated previously, Tau-seeding induced robust Tau-aggregation, however, quantitation of the aggregated Tau by IF revealed a striking increase of Tau-aggregation following additional seeding with Aβ (Fig. 2). Taken together, our data in the cellular Tau-aggregation assay indicate that Aβ-seeds can directly seed Tau-aggregation and can strongly accelerate propagation of pre-existing Tau-aggregates. The latter data are relevant in the context of spreading of pre-existing Tau-pathology from entorhinal cortex to functionally connected brain regions, driven by an increasing β-amyloid load. Pre-aggregated Aβ may thereby act as seeds to bypass or accelerate the lag-phase of Tau-aggregation by providing nucleation templates for Tau-aggregation.

### Pre-aggregated Aβ induces Tau fibrillization, in a cell-free assay, providing direct proof of heterotypic seeding

**Fig. 3:**
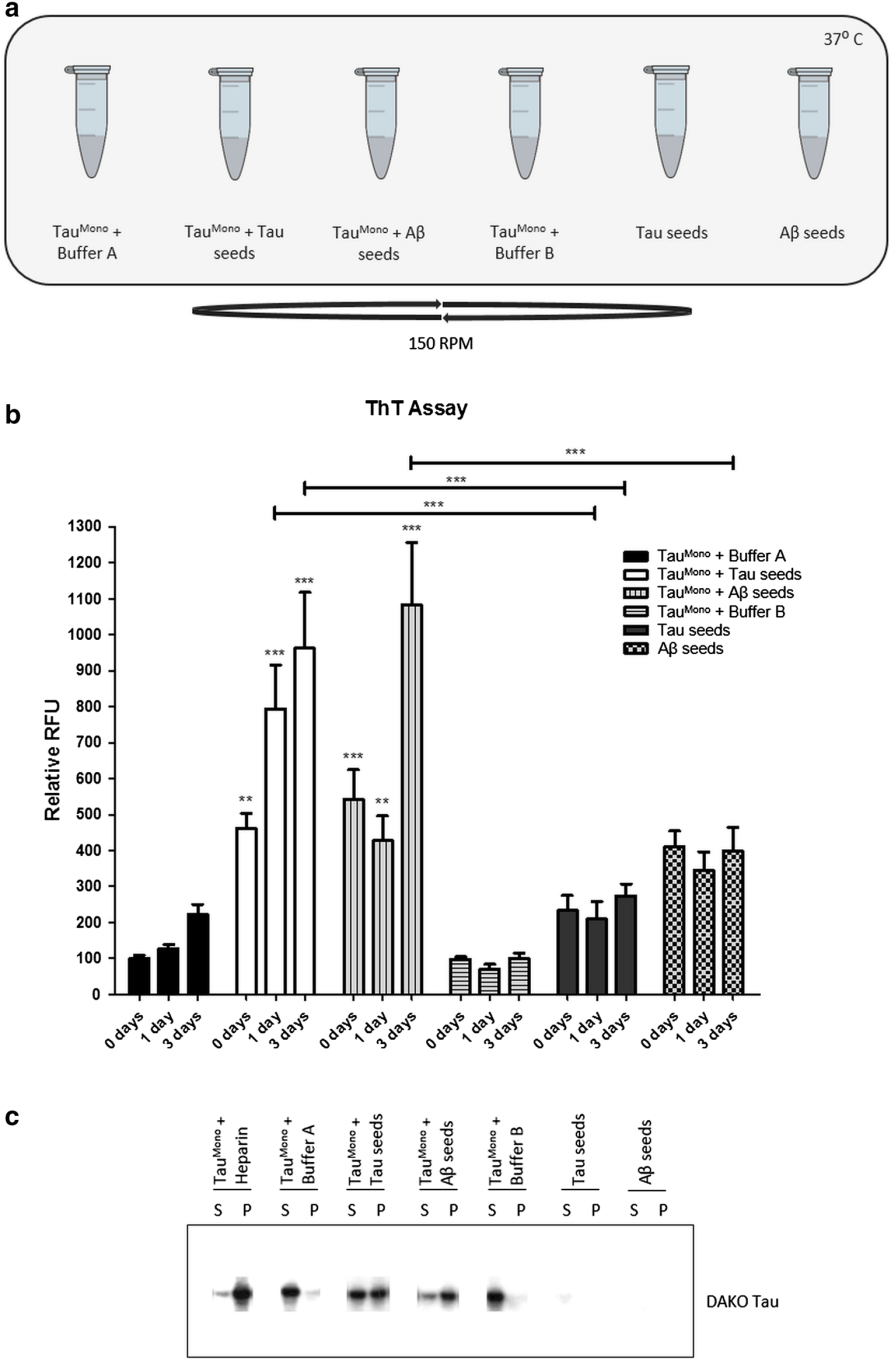
Pre-aggregated Aβ-seeds heterotypically seed aggregation of monomeric Tau in a cell-free assay, demonstrated by ThioT and sedimentation analysis. **a** Schematic presentation of the different experimental conditions of incubation. Synthetic pre-aggregated Aβ peptides, synthetic pre-aggregated Tau-seeds and their respective buffer conditions are co-incubated with monomeric Tau-fragments Tau^Mono^. **b** Aggregation of Tau^Mono^ by Tau-seeds and Aβ-seeds was measured using ThioT assay at 1 h, 1 day and 3 days post co-incubation. Quantitative analysis revealed significant induction of Tau-aggregation by Aβ-seeds and Tau-seeds (*n* = 9; two-way ANOVA followed by Bonferroni post hoc test; three independent experiments; mean ± SEM are presented; **p value <0.01, ***p value <0.001). **c** The presence of Tau-aggregates following co-incubation of Tau^Mono^ with either Tau-seeds or Aβ-seeds was demonstrated using a Tau-aggregates sedimentation assay, followed by Western blotting. Supernatant (S) and pellet (P) obtained following 100,000*g* centrifugation of co-incubated samples (3 days co-incubation) were analyzed by immunoblotting with Dako-Tau antibody

To further explore the hypothesis of direct induction of Tau-fibrillization by cross-seeding with pre-aggregated Aβ we used a cell-free assay similarly as previously used for demonstrating cross-seeding of alpha-synuclein and Tau [11]. We assessed if Aβ could directly cross-seed Tau-aggregation in a biochemical, cell-free assay (Fig. 3). Direct heterotypic seeding between pre-aggregated Aβ and Tau would be supported by previous data, demonstrating direct binding between Aβ peptides and Tau [18] and in silico models for Aβ-induced beta-sheet formation of Tau-fragments [34]. However, the final proof, i.e. Aβ-induced Tau fibril formation and its subsequent propagating potential, has not been demonstrated. In this biochemical assay Tau fibrillization is dependent on a rate-limiting nucleation-phase, which has been demonstrated to be strongly catalyzed by the presence of heparin and pre-aggregated synthetic Tau-fragments. Monomeric Tau-fragments (Tau^Mono^) were incubated at 37 °C under shaking conditions, and ThioT fluorescence was measured 1 h, 24 h and 3 days post-incubation with, respectively, heparin, pre-aggregated Tau-seeds, pre-aggregated Aβ-seeds, and the respective buffers of the seeds as control. Quantitative analysis at the different time points post-seeding revealed that Aβ-seeds induced significant Tau-aggregation at 3 days post-seeding (Fig. 3). Further controls were performed with albumin (a non-amyloidogenic protein) and with pre-aggregated amylin (a well-characterized unrelated amyloid protein) [9], revealing no significant induction of Tau-aggregation 3 days post-seeding (Fig. S3). Tau-aggregation induced by pre-aggregated Aβ-seeds was slower compared to induction by pre-aggregated Tau-seeds, but markedly and significantly increased compared to the buffer conditions (Fig. 3). This finding was further consistently reproduced in all subsequent experiments performed for follow-up experiments, since it was used as a primary control analysis of the samples. Next, direct Aβ-seeded Tau-aggregation was further confirmed using a sedimentation assay. Following incubation of the different conditions for 3 days, high-speed centrifugation was performed to pellet aggregated forms of Tau. Subsequently, pelleted and soluble forms of Tau were analyzed by Western blotting (Fig. 3). This analysis further confirmed the results obtained with ThioT, demonstrating that Aβ-seeds induced strong Tau-aggregation 3 days post-co-incubation.

**Fig. 4:**
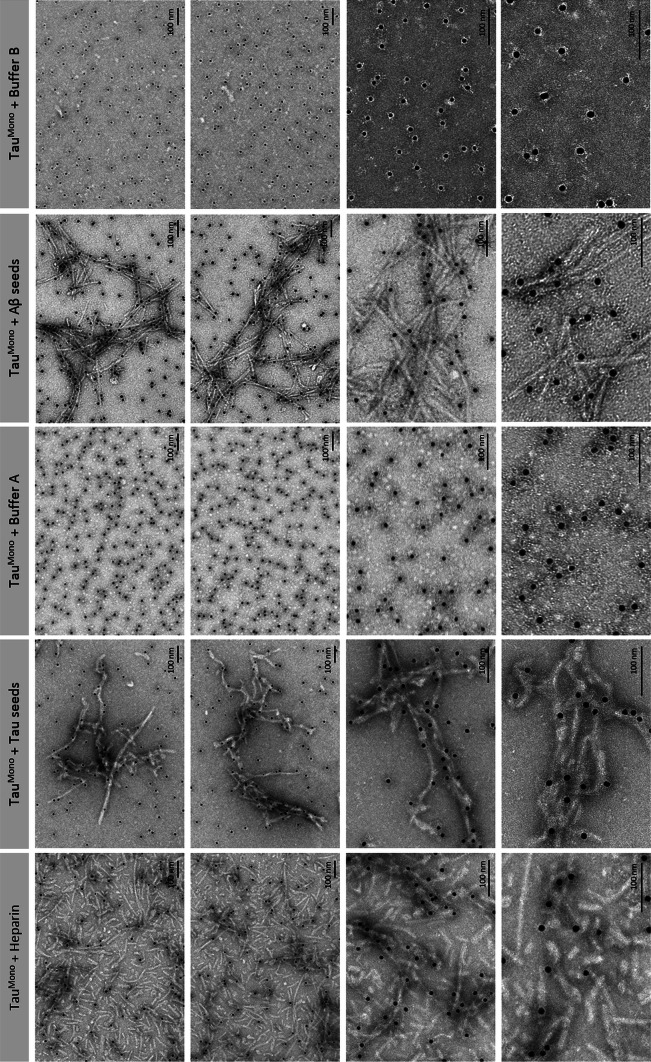
Pre-aggregated Aβ-seeds heterotypically seed fibrillization of monomeric Tau in a cell-free assay, demonstrated by immuno-electron microscopy analysis. The different conditions of co-incubation analyzed with the ThioT assay and sedimentation assay were subsequently analyzed by immuno-electron microscopy with anti-Tau antibody (DAKO-Tau; 10 nm-gold labeled). This demonstrated strong induction of Tau fibrillization following direct co-incubation of Tau-seeds with Tau^Mono^ as demonstrated previously. Co-incubation of Aβ-seeds with Tau^Mono^ very strongly resulted in Tau-fibrillization. Incubation of Tau^Mono^ with the respective buffers did not induce Tau-fibrils; only globular structures were detected. Higher magnifications of Aβ-seed-induced Tau fibrils and Tau-seed-induced Tau fibrils are presented (*scale bar* 100 nm)

To analyze the structure of Aβ-seeded Tau-aggregates, we performed negative-staining electron microscopic analysis (EM) in combination with immuno-EM using a Tau-specific antibody 3 days post-incubation. This revealed the abundant presence of 10- to 15-nm wide fibrillar Tau aggregates in samples incubated with heparin, pre-aggregated Tau-seeds and pre-aggregated Aβ-seeds, while filaments were nearly absent in buffer only conditions (Fig. 4). Strong induction of fibrillar Tau-aggregates was demonstrated following induction of Tau-aggregation with heparin. Fibrillar Tau-aggregates were also abundantly detected following seeding with pre-aggregated synthetic Tau-seeds, yet less strong as with heparin (Fig. 4). This is reflected in the fact that non-fibrillar and smaller globular Tau-forms were also still detected. Importantly, incubation with Aβ-seeds induced abundant formation of Tau-fibrils to comparable extents as following addition of Tau-seeds (Fig. 4). These fibrils were identified by staining with immuno-EM as Tau-fibrils, using a Tau-specific antibody. The specificity of this staining was demonstrated by appropriate control stainings, with buffer only (no Tau) and without primary antibody revealing no staining (Fig. S4). Furthermore, increasing dilutions of Tau, demonstrated decreasing staining with lower concentrations, further highlighting the specificity of the staining (Fig. S4). These data clearly demonstrate that pre-aggregated Aβ can seed filamentous Tau-aggregation following direct co-incubation. These data thereby prove heterotypic seeding of filamentous Tau aggregation by Aβ.

**Fig. 5:**
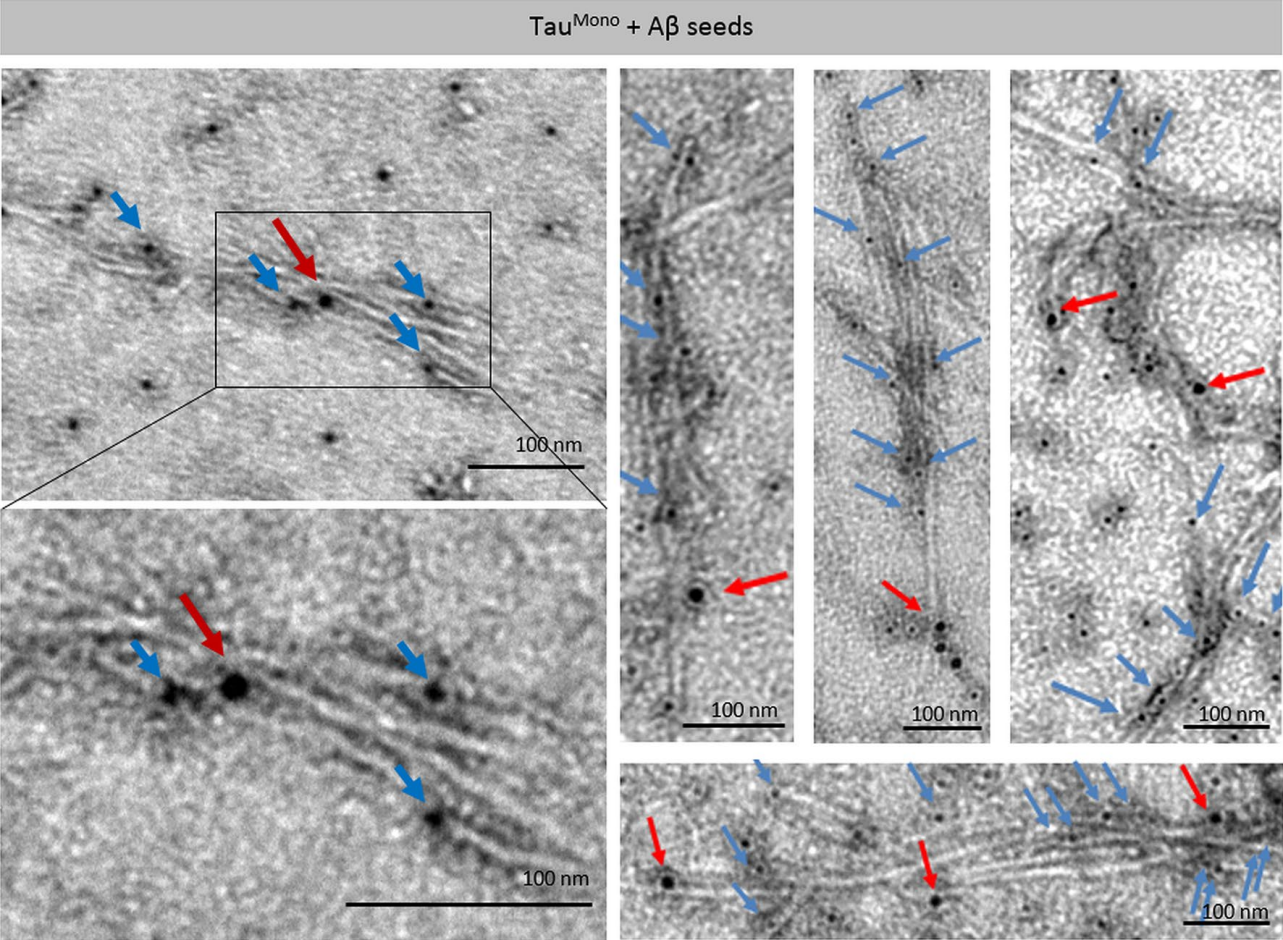
Pre-aggregated Aβ provides seeds for Tau-fibrillization in a cell-free assay, demonstrated by immuno-electron microscopy analysis. Double-immuno-EM analysis using anti-Aβ antibody (WO2; 10 nm-gold labeled; red arrows) and anti-Tau antibody (DAKO-Tau; 5 nm-gold labeled, blue arrows), reveals the presence of Aβ within Tau-filaments. Aβ is scarcely detected per fibril compared to the abundant detection of Tau, suggesting that Aβ-seeds provide a nucleus, which is subsequently preferably extended by Tau^Mono^ (*scale bar* 100 nm)

We next performed double immuno-EM analysis using anti-Aβ and anti-Tau antibodies, to demonstrate cross-seeding. This clearly demonstrated the combined presence of Aβ and Tau in the Aβ-induced Tau-filaments, demonstrating cross-seeding of Tau by pre-aggregated Aβ (Fig. 5). Specificity of the Aβ staining was demonstrated by strong specific staining of pre-aggregated Aβ and appropriate negative controls (buffer only and no primary antibody, revealing absence of staining) (Fig. S4). Using this staining, anti-Aβ-stained structures were detected at limited spots within or at the basis of the Tau-stained filaments (Fig. 5). This is in line with Aβ-seeds providing a nucleus for subsequent Tau-fibril formation. Following Aβ-induced seeding the Tau-filaments seem to be ‘preferentially’ elongated by Tau rather than Aβ.

### Heterotypic Aβ-seeded Tau provides potent seeds for induction and propagation of Tau-pathology in vitro and in vivo

**Fig. 6:**
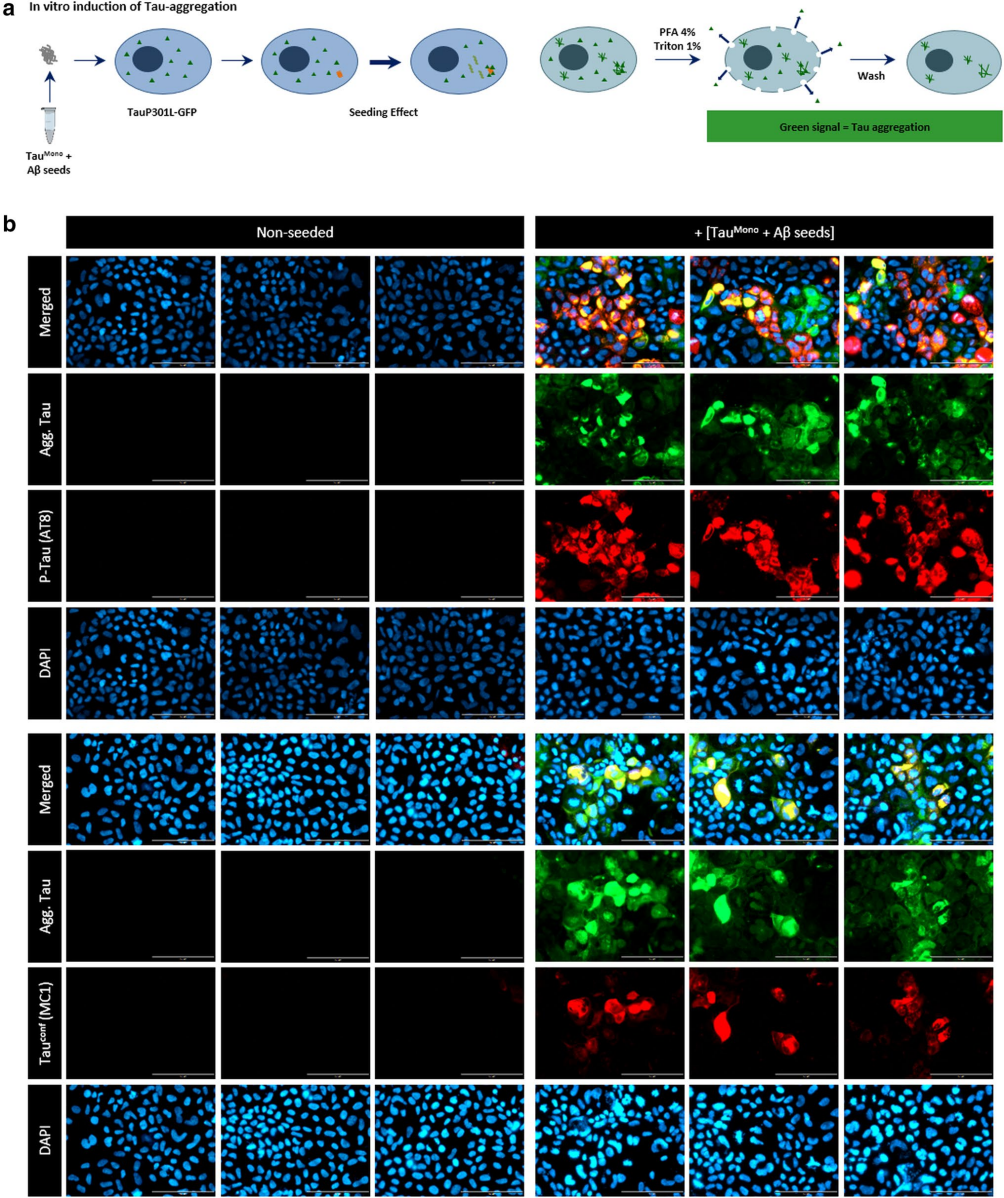
Aβ-induced Tau-aggregates provide potent seeds for prion-like Tau-aggregation in cells in vitro. **a** Schematic presentation of the assay. Induction of Tau-aggregation was performed using heterotypic Aβ-seeded Tau^Mono^ as “seeds” in TauP301L-GFP expressing cells, to analyze their propagating potential. Analysis is performed by cytological analysis following stringent detergent extraction to eliminate soluble forms of Tau. **b** This analysis demonstrated efficient induction of Tau-aggregation following seeding with Aβ-seeded Tau^Mono^, reflected in strong induction of aggregated Tau, phosphorylated at pathologically relevant epitopes (AT8) and conformationally altered (MC1). Representative images of GFP-signal following stringent extraction with 1 % TX-100 and immunofluorescent staining using antibodies recognizing phosphorylated Tau (S202/T205) (AT8) and conformationally altered Tau (MC1) are presented, in non-seeded conditions and following seeding with Aβ-seeded aggregated Tau (*scale bar* 100 µm)

Having demonstrated that pre-aggregated Aβ can heterotypically seed Tau filament formation, we analyzed the potential of these Aβ-induced Tau-aggregates to seed Tau-pathology in vitro. Aβ-seeded Tau-aggregates were used to seed Tau-aggregation in the above described Tau-aggregation assay in QBI-293 cells (Fig. 6). This revealed the potential of Aβ-induced Tau-seeds (further denoted as Aβ-seeded Tau) to seed Tau-pathology in the cellular Tau-aggregation assay. Tau-aggregates induced by Aβ-seeded Tau, strongly stained with AT8 and MC1 antibodies, revealing the presence of hyperphosphorylated, conformationally altered Tau aggregates. Comparative quantitative analysis revealed a significantly higher induction of Tau-aggregation compared to homotypic Tau-seeds, further highlighting that these seeds efficiently induce Tau-aggregation in vitro (Fig. S5).

**Fig. 7:**
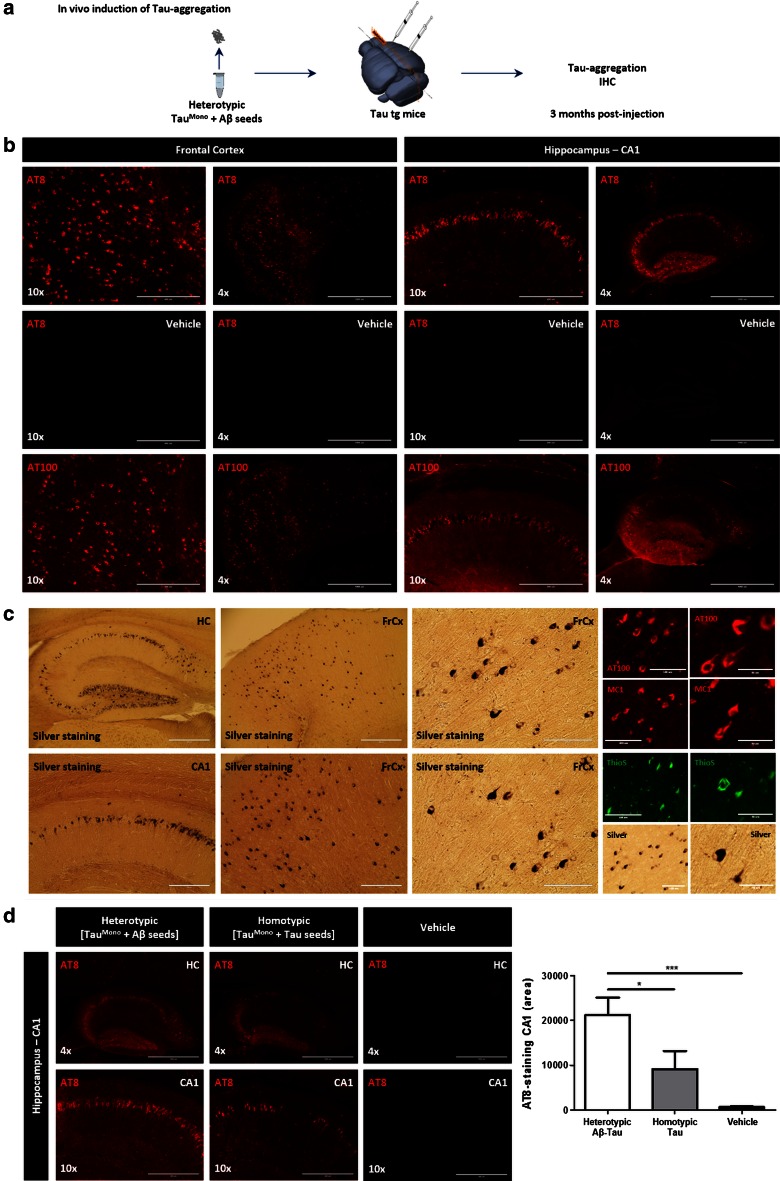
Heterotypic Aβ-seeded Tau efficiently induces Tau-aggregation in Tau transgenic mice in vivo. **a** Schematic presentation of the assay: Induction of Tau-aggregation using heterotypic Aβ-seeded Tau^Mono^ as “seeds” in Tau transgenic mice. Tau transgenic mice (4 months old) were intracerebrally injected in frontal cortex and hippocampus and analyzed 3 months post-injection using AT8 to assess induction of Tau-pathology in frontal cortex and hippocampus. **b** Representative images of AT8 and AT100 staining in frontal cortex and hippocampus are shown following injection with heterotypic Aβ-seeded Tau^Mono^ (further denoted heterotypic Aβ-seeded Tau) (4× and 10× magnifications are shown, *scale bar* 1000 and 400 μm, respectively). No AT8 staining was detected in vehicle injected mice, following concomitant staining and imaging. **c** Tau-pathology induced by Aβ-seeded Tau is strongly stained with AT8, AT100, MC1, Gallyas silver staining and ThioS staining. This demonstrates the true fibrillar nature of NFTs induced by Aβ-seeded Tau injections. Concomitant stainings with AT8, AT100, MC1 and silver staining in vehicle injected mice revealed no staining in Tau transgenic mice, 3 months post-injection (data not shown). **d** Comparative analysis of seeding efficiency of heterotypic Aβ-seeded Tau and homotypic Tau-seeds in Tau transgenic mice at 3 months post-injection. Quantitation of AT8 staining demonstrated increased AT8 staining following injections with heterotypic Aβ-seeded Tau compared to homotypic Tau-seeds, while Tau-pathology was significantly induced in both conditions compared to vehicle injected mice (*n* = 6 mice per condition; one-way ANOVA followed by Tukey’s post hoc test; mean ± SEM are presented; *p value <0.05, ***p value <0.001)

We next analyzed the seeding potential of Aβ-seeded Tau in vivo by injection in TauP301S transgenic mice [58], using a similar approach as previously published by us and others [22, 51]. Due to the procedure of heterotypic seeding used in this work, five times more diluted final concentrations of Tau-seeds needed to be used compared to our previous work [51]. Induction of Tau-aggregation was analyzed 3 months post-injection following intracerebral injections in frontal cortex and hippocampus, using optimized protocols in our group [39, 51]. Our analysis indicated a strong induction of Tau-pathology by heterotypic Aβ-seeded Tau compared to non-seeded monomeric Tau or buffer injections only, 3 months post-injection (Fig. S6). We furthermore compared these newly identified heterotypic Aβ-seeded Tau seeds with homotypic Tau-seeds, which have been shown to be potent inducers of Tau-aggregation and propagation of Tau-pathology in transgenic mice in vivo [51]. Quantitative analysis revealed that Aβ-seeded Tau even more potently induced Tau aggregation than homotypic Tau-seeds (Fig. 7), thereby highlighting the high potency and efficiency of these seeds to induce Tau-aggregation in vivo. Tau-aggregates induced by Aβ-seeded Tau seeds were stained with antibodies recognizing pathological forms of Tau, i.e. AT8, MC1 and AT100. Tau-aggregates were furthermore stained following Gallyas silver staining and ThioS staining, revealing the true fibrillary nature of these aggregates. Aβ-staining did not reveal any signal, demonstrating that these aggregates are mainly composed of Tau.

**Fig. 8:**
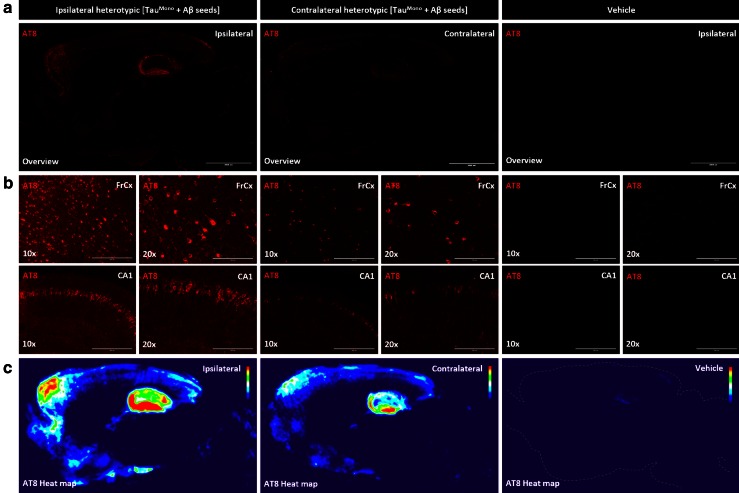
Aβ-seeded Tau propagates Tau pathology along functional connections in Tau transgenic mice in vivo. **a** Representative AT8 stainings of sagittal brain sections of heterotypic Aβ-seeded Tau in frontal cortex and hippocampus (FrCx/HC), from the ipsi-lateral (injected) hemisphere and the contralateral (non-injected) hemisphere in comparison with vehicle injected brains of Tau transgenic mice 3 months post-injection, are shown (*scale bar* 2000 μm). **b** Higher magnifications of frontal cortex and hippocampal regions of the ipsi-lateral side and contralateral side, of mice injected with heterotypic Aβ-seeded Tau in frontal cortex and hippocampus and vehicle injected Tau mice are presented (10×, 20× magnifications are shown; *scale bar* 400 and 200 μm, respectively). This reveals clear-cut induction of Tau-pathology, at the contralateral side of heterotypic Aβ-seeded Tau injected mice, compared to vehicle injected brains, but less strong compared to the injected side, demonstrating propagation of Tau-pathology to remote but functionally connected brain regions. Tau-pathology stained with AT8 correlates strongly with Gallyas silver staining and ThioS (results not shown). **c** To analyze propagation of Tau pathology semi-quantitatively, averaged heat maps representing color-scored, averaged images of AT8 stained Tau-pathology following injection of heterotypic Aβ-seeded Tau in frontal cortex and hippocampus (FrCx/HC) and vehicle injections were generated. Averaged heat maps of AT8 stained Tau-pathology at the side of injection (denoted “ipsi-lateral”) and the contralateral non-injected side (denoted “contralateral”) were generated. Heat maps were generated by averaging AT8 stainings of multiple mice for each injection paradigm using Image J (*n* = 6 for each condition). This reveals clear-cut induction of averaged Tau-pathology in the non-injected hemisphere of heterotypic Aβ-seeded Tau-injected brains (in frontal cortex and hippocampus), absent in age-matched vehicle injected mice, but less strong compared to the injected brain hemisphere, demonstrating propagation of Tau-pathology to remote but functionally connected brain regions

To further assess if Aβ-seeded Tau-seeds induced spreading or propagation of Tau-pathology, we performed an immunohistochemical analysis of Tau-pathology of the contralateral side, following unilateral seeding. We have previously demonstrated spreading of Tau-pathology following injections in different brain regions, i.e. entorhinal cortex, basal ganglia and combined hippocampal/cortical injections, to functionally connected brain regions [51]. We here demonstrate that unilateral injection of Aβ-seeded Tau-seeds resulted in propagation of Tau-pathology to the contralateral side. Tau-pathology was significantly induced compared to vehicle injected mice, but remained less prominent compared to the ipsi-lateral side, as demonstrated by a semi-quantitative heat-map analysis (Fig. 8) and by quantitative analysis (Fig. S7), in line with propagation from the injected side. Together, our data indicate that Aβ-seeded Tau provides strong seeds for induction and propagation of Tau-pathology.

Together, our data demonstrate heterotypic seeding of filamentous Tau-aggregation by pre-aggregated Aβ. Aβ-seeds thereby provide seeds for Tau-aggregation, strongly accelerating the lag-phase for Tau-aggregation. These newly identified heterotypic Aβ-seeded Tau-seeds provide potent seeds for induction and propagation of Tau-pathology in vivo.

## Discussion

In this work we have demonstrated (1) direct seeding of Tau-fibrillization by pre-aggregated Aβ in a cell-free assay, demonstrating filamentous Tau-aggregation by heterotypic seeding with Aβ-seeds. We furthermore demonstrate (2) induction of Tau-aggregation and acceleration of incipient Tau-aggregation by Aβ-seeds in a well-characterized cellular assay of Tau-aggregation and (3) finally we analyzed the repercussions of these heterotypic Aβ-seeded Tau in vitro and in vivo. We thereby demonstrate that heterotypic seeded Tau by Aβ provides potent seeds to induce and propagate Tau-aggregation in vivo. Taken together, we demonstrate that pre-aggregated Aβ can template filamentous Tau aggregation by cross-seeding, providing potent seeds for induction and propagation of Tau-pathology in a prion-like way in vivo. This presents a compelling molecular mechanism of Aβ-induced propagation of Tau-pathology in AD.

In this work, we demonstrate direct seeding of Tau-fibrillization by pre-aggregated Aβ in a cell-free assay. Prion-like seeding mechanisms have gained important interest as pathogenetic mechanism in a variety of neurodegenerative proteinopathies [6, 8, 12, 16, 17, 22, 27, 28, 30, 33, 38, 39, 51, 56]. Homotypic seeding and prion-like properties have been demonstrated for different proteins associated with these neurodegenerative disorders [6, 8, 12, 16, 17, 22, 27, 28, 30, 33, 38, 39, 51, 56] including Tau. Heterotypic seeding or cross-seeding has been demonstrated for Tau and alpha-synuclein, and for different combinations of amyloidogenic proteins in vitro [11, 14, 15, 35]. Direct cross-seeding between pre-aggregated Aβ and Tau is supported by demonstrating direct binding between Aβ peptides and Tau [18] and in silico models for Aβ-induced beta-sheet formation of Tau-fragments [34]. However, we here demonstrate direct induction of Tau fibrillization by pre-aggregated Aβ-seeds, in an unequivocal way using ThioT assay, sedimentation analysis and immuno-EM. Having demonstrated the induction of Tau-fibrillization in a cell-free assay, we further extended this analysis in a well-characterized cellular Tau aggregation assay. In this assay, the nucleation-dependent initial lag phase for Tau-fibrillization in cells is proposed to be by-passed by the addition of preformed pre-aggregated synthetic Tau-seeds, which accelerate fibrillization of monomeric Tau. This demonstrated that pre-aggregated Aβ was able to seed Tau-aggregation, similarly as Tau-seeds, albeit less efficient. Previous data demonstrated the occurrence of homotypic seeding for Aβ, alpha-synuclein, Tau and huntingtin in a cellular Tau aggregation assay, while heterotypic cross-seeding was not observed [44]. The observed difference in our current data-set may rely on the fact that homotypic seeding is much more efficient compared to heterotypic seeding, thereby masking the effect of heterotypic seeding in the previous study which was focused on homotypic seeding, or may rely on the properties of the Aβ-seeds used or assay sensitivity. In favor of this is the demonstration of heterotypic seeding of alpha-synuclein and Tau, in a similar Tau-aggregation assay and in primary neurons [15, 57], further corroborating the potential of heterotypic seeding between prion-like proteins in cellular assays. Clearly, in our assay heterotypic seeding was much less efficient compared to homotypic seeding. Interestingly, Aβ-seeds potently induced Tau-aggregation in the cellular Tau-aggregation assay, when incipient Tau-aggregates were present. This finding is reminiscent and intriguing in the context of AD, as outlined below. Furthermore, inflammatory processes or neuron-specific mechanisms can be mechanistically excluded for Aβ-induced Tau-aggregation in this assay, since a non-neuronal cell line is used. Our assay suggests that pre-aggregated Aβ-seeds provide seeds for templating Tau-aggregation, bypassing the required nucleation phase for Tau-aggregation. Neither monomeric Aβ nor monomeric Tau forms induced Tau-aggregation in this assay, further strengthening templated seeding as the mechanism involved. Finally, we demonstrated that heterotypically aggregated Tau by Aβ, provided potent and efficient seeds for induction and propagation of Tau-aggregation in vivo in Tau transgenic mice. Tau-pathology induced by Aβ-seeded Tau was characterized by immunostaining with antibodies recognizing Tau phosphorylated at pathologically relevant epitopes, i.e. AT8, AT100 and recognizing conformationally altered Tau. Tau aggregates were stained by Gallyas silver staining and ThioS staining, demonstrating the true fibrillary nature of these aggregates. Tau pathology propagated efficiently from the injected brain hemisphere to the contralateral side, in a similar pattern as previously observed [39, 51]. Our data thereby demonstrate the strong potential of heterotypic Aβ-seeded Tau to seed and propagate Tau-pathology in vivo, strengthening the relevance of these newly identified seeds.

**Fig. 9:**
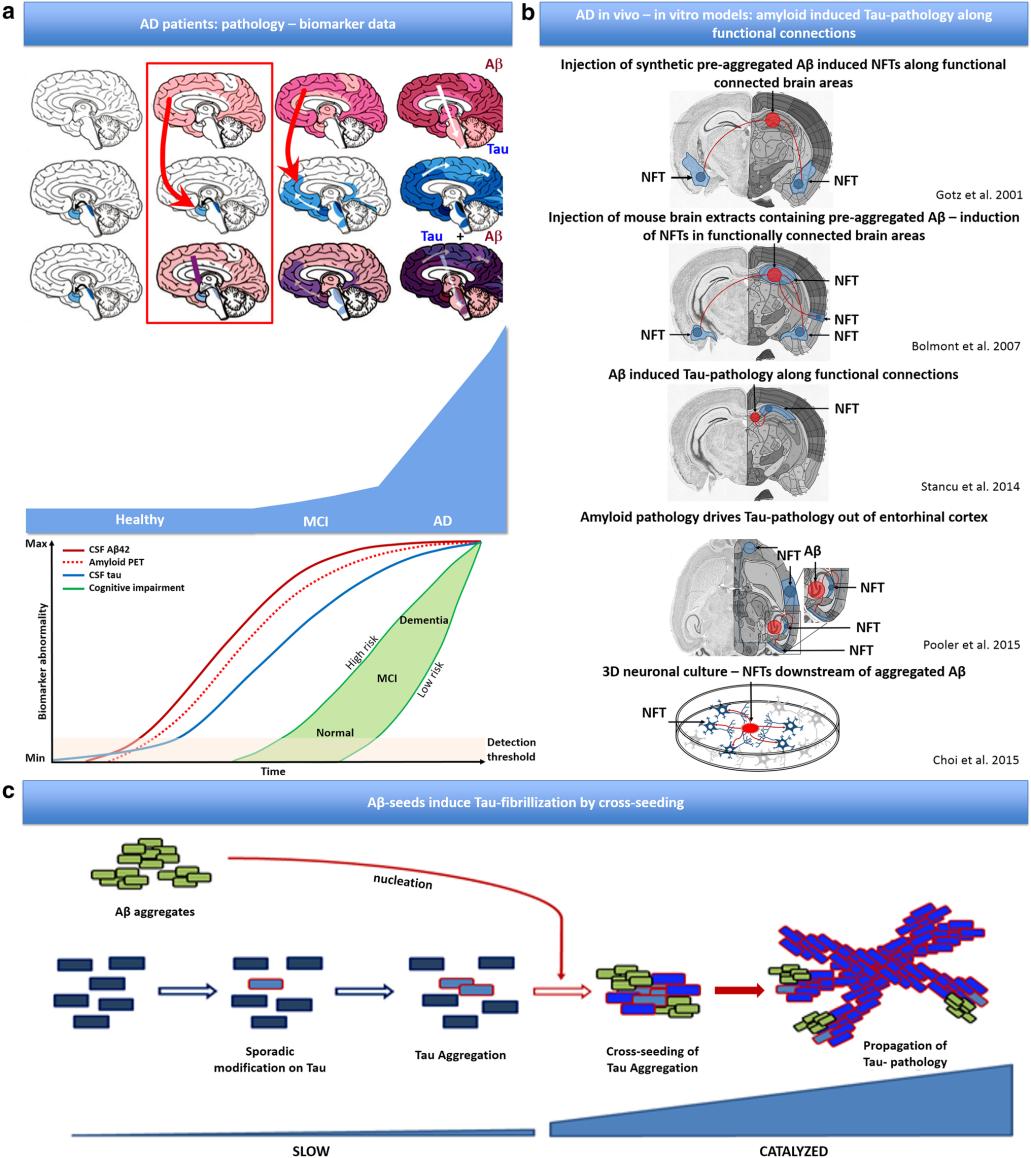
Schematic summary of the work. **a** Clinical, genetic, pathological and biomarker data in patients support amyloid induced Tau-pathology and subsequent propagation beyond EC as a crucial process in the conversion from preclinical stages to AD. Schematic views are reproduced with minor modifications from publications of Jucker and Walker 2013 [27] and Jack et al. 2013 [25]. **b.** Data from different in vivo and in vitro models support Aβ induced Tau-pathology to functionally connected brain regions following injections with (1) pre-aggregated synthetic Aβ and (2) pre-aggregated Aβ from brain extracts from amyloid plaque displaying AD models and (3) AD patients, as well as in different preclinical AD models displaying Aβ induced Tau-pathology. Schematic presentations of the results presented in previous papers are presented [2, 5, 13, 40, 50]. This process is reminiscent of the spatial dissociation of Aβ- and Tau-pathology in AD, in which (pre-) aggregated Aβ aggravates Tau-pathology along functional connections [3, 54]. These findings are reminiscent of the prion-like propagation of Tau-pathology along functionally connected brain regions in Tau-models. Aβ-induced Tau-pathology could hence be driven by cross-seeding between Aβ-seeds and Tau, similarly as proven for alpha-synuclein and Tau. **c** In this work, we demonstrate that pre-aggregated Aβ provides seeds for Tau-fibrillization by cross-seeding, providing potent seeds for inducing Tau-aggregation and propagation of Tau-pathology in Tau-transgenic mice in vivo. Without excluding involvement of other mechanisms, including inflammation and synaptic/neuronal processes, Aβ-induced Tau-aggregation by cross-seeding provides a compelling molecular model for Aβ induced Tau-pathology in preclinical models of AD and in AD patients, capable of resolving some particularities observed in AD patients—as outlined in “Discussion”

Our data indicate a compelling mechanism for Aβ-induced Tau-pathology in preclinical models of AD. Since Aβ-induced Tau-pathology is situated at the effective beginning of AD, i.e. the conversion from preclinical stages to MCI and subsequently to AD, it is considered as a key-pathogenetic mechanism. Its understanding is crucial to understand AD pathogenesis and design of therapeutic strategies [3, 19, 25, 26, 46, 47, 53, 54]. Aβ-induced Tau-aggregation has been reproducibly and robustly recapitulated in a variety of different in vivo preclinical models using different experimental approaches [2, 13, 20, 21, 29, 36, 40, 45, 49, 50]. Thus, Aβ-induced Tau-pathology was demonstrated by (1) crossing or co-expressing of mutant APP/PS1 with mutant Tau in transgenic mice [2, 29, 36, 40, 49] following (2) injection of pre-aggregated Aβ containing extracts derived from AD patients or APP/PS1 transgenic mice [2], or (3) following injection of synthetic pre-aggregated Aβ [13]. Different cellular and molecular mechanisms must be considered for Aβ-induced Tau-pathology in these models, including neuron-specific and synaptic/synaptotoxic mechanisms [13], or a role of Aβ-induced inflammation and other potential mechanisms [13, 23, 24, 48, 49]. Our current data demonstrating that Aβ-seeds can heterotypically induce filamentous Tau-aggregation, providing potent seeds for induction of Tau-aggregation, demonstrate an attractive mechanism for Aβ-induced Tau-aggregation in these preclinical in vivo models. Interestingly, in these preclinical models pre-aggregated Aβ strongly induced Tau-pathology at the site of injection but also in remote brain regions functionally connected to the injection site [2, 13, 40, 50] (Fig. 9). The induction and spreading of Tau-pathology in these preclinical models in functionally connected brain regions is reminiscent of prion-like Tau-seeding [1, 7, 22, 27, 51]. Tau-pathology is thereby propagated to remote but functionally connected brain regions, affecting intrinsic functional neuronal networks, reminiscent of the networks affected in AD and related Tauopathies [1, 22, 51]. We here propose heterotypic seeding of Tau by Aβ as a contributing mechanism of Aβ-induced Tau-pathology in these preclinical models. It must be noted that prion-like seeding is mechanistically not yet fully resolved, in terms of uptake and spreading of “seeds”, nor for Tau-seeds, nor in the case of cross-seeding for Aβ-seeds. Nevertheless, prion-like seeding has been reproducibly recapitulated in different models using different types of “seeds”. Taken together, the heterotypic seeding of filamentous Tau aggregation, along functional connections, providing potent seeds for Tau-aggregation as demonstrated in this work, presents as an attractive molecular mechanism of Aβ-induced propagation of Tau pathology, in functionally connected brain regions as observed in preclinical AD models (Fig. 9).

Heterotypic seeding of Tau-pathology by Aβ-seeds yielding potent seeds for propagation of Tau pathology in vivo, as demonstrated in this work, strongly fits with published genetic, pathological, clinical and biomarker data from patients. It presents as a compelling mechanism for Aβ-induced Tau-pathology in AD and fits some peculiar findings in AD patients. Tau-pathology in EC and LC is present already early in life in most healthy people, but this early pathology does not spread beyond EC and LC and is not associated with clinical symptoms [3, 4, 19, 25, 26, 31, 46, 47, 53, 54]. Hence, this Tau-pathology can be considered as rather “silent” or “non-aggressive” (yet a form of pre-existing pathological Tau). In AD, pathological analysis, clinical analysis and biomarker data indicate that following accumulation of a high β-amyloid load in isocortical regions, Tau-pathology is propagated beyond EC/LC in a more aggressive way and with associated symptoms [3, 4, 19, 25, 26, 31, 46, 47, 53, 54]. Heterotypic seeding of Tau-pathology by Aβ-seeds thereby provides a compelling molecular mechanism of Aβ-induced propagation of Tau-pathology in AD, capable of resolving some peculiar findings in AD as further outlined.

Firstly, in AD Aβ and Tau-pathology progress in remarkably distinct spatio-temporal patterns according to Thal and Braak staging [3, 54]. Nevertheless, increasing Aβ load in remote brain regions is generally accepted to drive propagation of Tau-pathology. Heterotypic induced Tau-aggregation by Aβ-seeds is in line with this (1) spatial paradox, since this seeding process can occur along functional connections. Hence, Aβ-seeds, strongly accumulating in isocortical regions can via functional connections cross-seed Tau-pathology in EC to spread beyond EC. Prion-like seeding and propagation of Tau-aggregation has been demonstrated to occur between functionally connected brain regions [1, 6, 16, 17, 22, 28, 39, 44, 51], reminiscent of the progression of Tau-pathology in AD [1, 3, 7, 22, 27, 51]. Interestingly, in vivo models of Aβ-induced Tau-pathology display features reminiscent of this prion-like seeding process [2, 13, 40, 50].

Secondly, it is intriguing that in AD brains, despite accumulation of high β-amyloid plaque load in isocortical brain regions, these regions remain (2) relatively spared from Tau-pathology in earlier stages of the disease [3, 54]. And instead, there is a (3) preferential propagation of Tau-pathology out of regions with pre-existing Tau-pathology, resulting in the very characteristic Braak staging. Intriguingly, we here demonstrate that Aβ-seeded Tau-aggregation efficiently accelerates pre-existing Tau-pathology. This is also in line with findings in preclinical models, where no induction of Tau-aggregation is demonstrated in models expressing APP/PS1, or following injection of pre-aggregated Aβ without overexpression of Tau on the murine Tau background, in contrast to models overexpressing mutant Tau [49].

Thirdly, heterotypic seeding of Tau by Aβ could explain (4) conversion to more potent and aggressively propagating Tau-pathology beyond EC, following cross-seeding by Aβ-seeds. And finally, since (5) homotypic prion-like seeding occurs preferentially, initially Aβ load will increase preferentially and exponentially in isocortical regions [54]. This exponentially increasing Aβ load may thereby increase dramatically the probability of cross-seeding, inducing propagation of Tau-pathology, associated with the conversion to AD.

Taken together, heterotypic seeding of filamentous Tau-aggregation by Aβ presents as a compelling mechanism for Aβ-induced Tau-pathology, capable of resolving some peculiar AD patient data. However, we do not exclude the potential involvement of other molecular and cellular processes in Aβ-induced Tau-pathology, particularly neuronal signaling pathways and inflammation. The respective contribution of these mechanisms to Aβ-induced Tau-pathology in preclinical AD models and AD patients remains to be further analyzed.

In conclusion, Aβ-induced Tau-pathology is based on genetic data, biomarker data, clinical data and data from in vitro and in vivo models to be considered as a key initiating mechanism in the pathogenesis of AD, more particularly in the conversion from preclinical stages to MCI and AD [3, 4, 19, 25, 26, 31, 46, 47, 53, 54]. Unraveling the exact molecular mechanisms which can actually contribute to Aβ-induced Tau-pathology is required for design of novel therapeutic venues to halt or slow down conversion to AD. In this work we have proven the occurrence of heterotypic seeding of filamentous Tau-aggregation by Aβ and further demonstrated the seeding and propagating potential of these newly identified species in vivo. We believe this presents a very compelling mechanism for Aβ-induced Tau-pathology in preclinical models of AD, and AD patients, which is in line with and capable of resolving some characteristic findings in AD patients.

## Electronic supplementary material

Supplementary material 1 (TIFF 42098 kb)

Supplementary material 2 (TIFF 62978 kb)

Supplementary material 3 (TIFF 60280 kb)

Supplementary material 4 (TIFF 65714 kb)

Supplementary material 5 (TIFF 62931 kb)

Supplementary material 6 (TIFF 38713 kb)

Supplementary material 7 (TIFF 15001 kb)

Supplementary material 8 (TIFF 18700 kb)

Supplementary material 9 (DOCX 25 kb)
