# Supplementary figures and images for: Heterotypic seeding of Tau fibrillization by pre-aggregated Abeta provides potent seeds for prion-like seeding and propagation of Tau-pathology in vivo
Source: Acta Neuropathol. 2016 Jan 6;131:549–69. doi: 10.1007/s00401-015-1525-x (PMC4789256; doi:10.1007/s00401-015-1525-x)

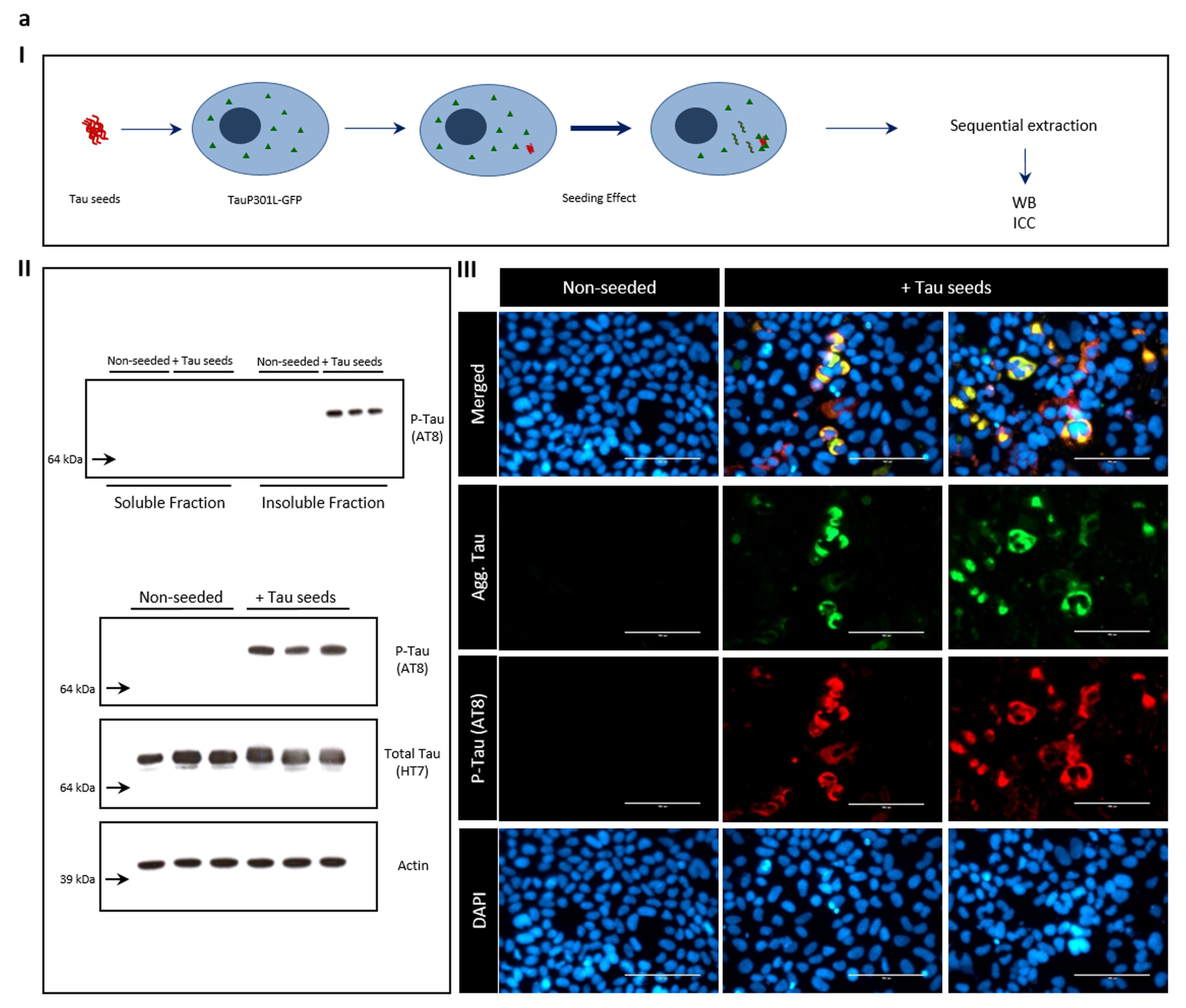

Supplement: Supplementary file 1 — Supplementary material 1 (TIFF 42098 kb) [file 401_2015_1525_MOESM1_ESM.tif]

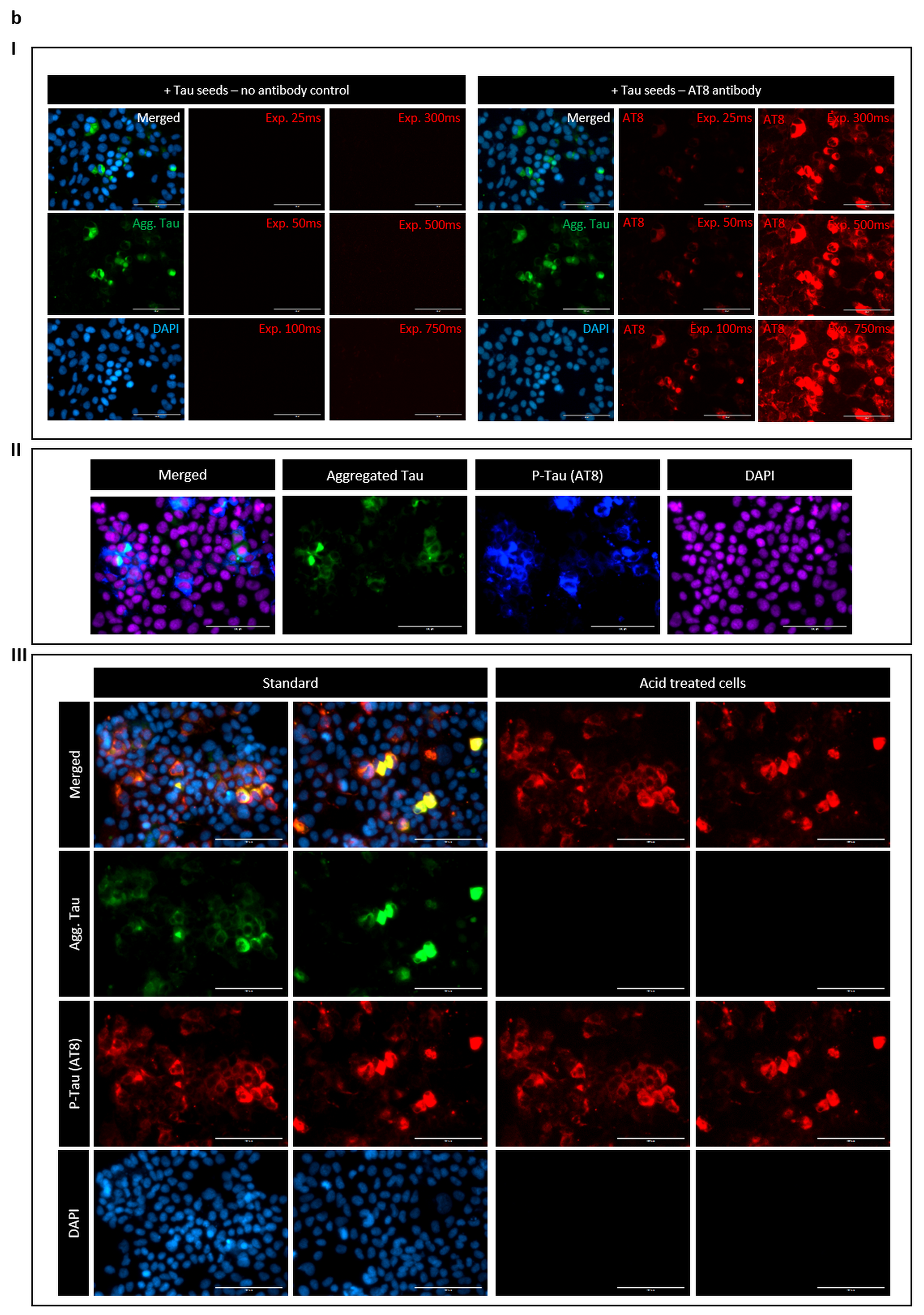

Supplement: Supplementary file 2 — Supplementary material 2 (TIFF 62978 kb) [file 401_2015_1525_MOESM2_ESM.tif]

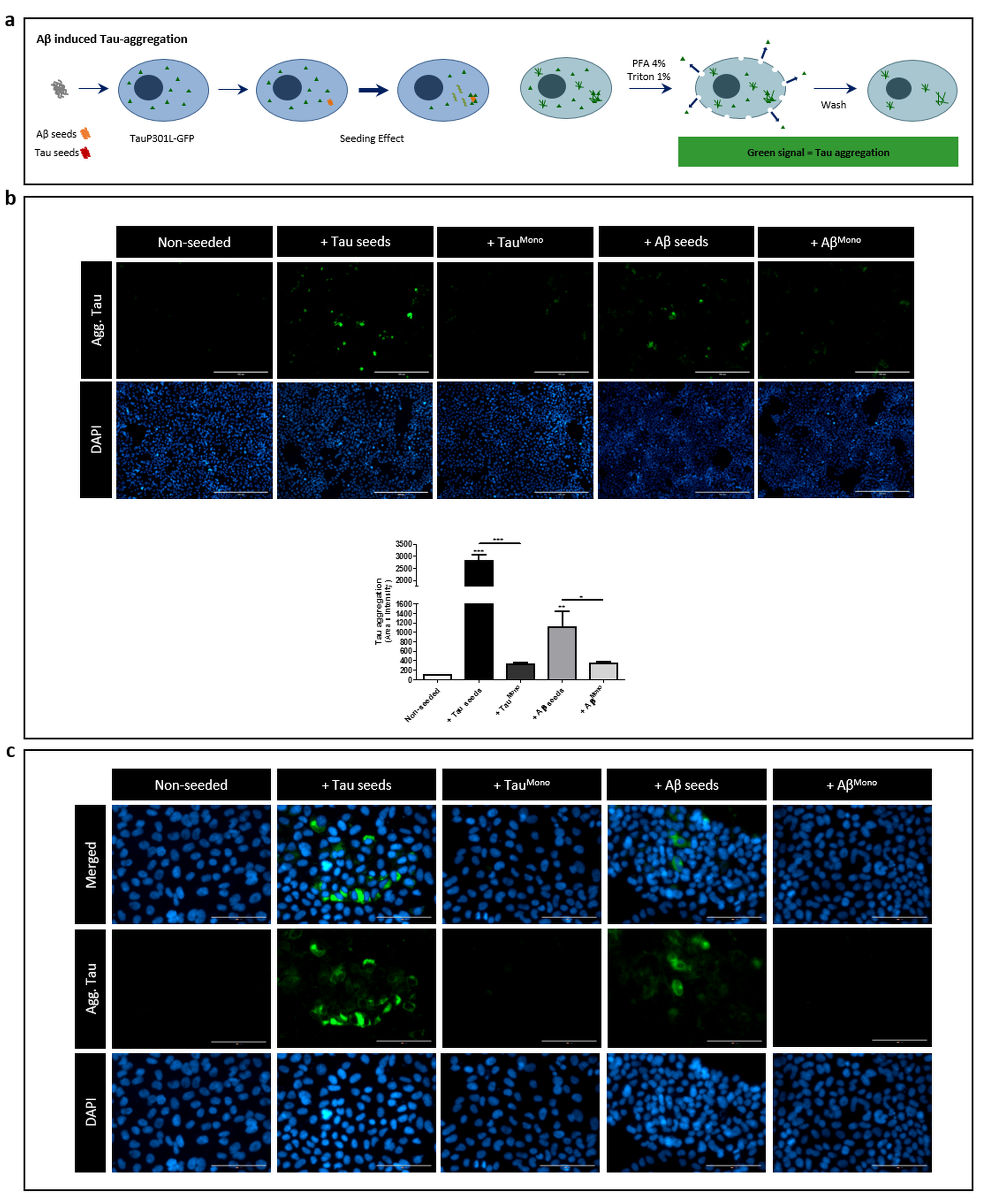

Supplement: Supplementary file 3 — Supplementary material 3 (TIFF 60280 kb) [file 401_2015_1525_MOESM3_ESM.tif]

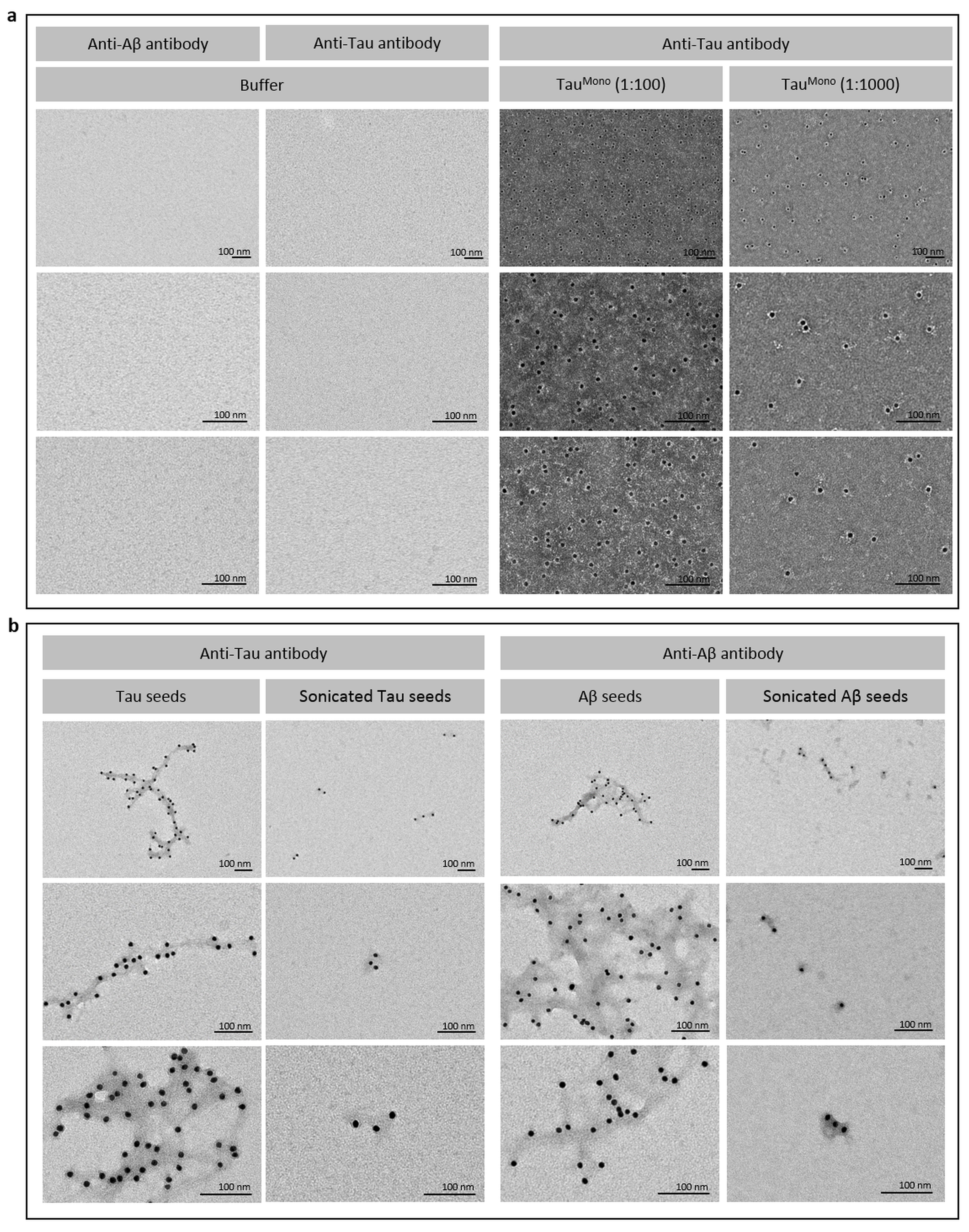

Supplement: Supplementary file 5 — Supplementary material 5 (TIFF 62931 kb) [file 401_2015_1525_MOESM5_ESM.tif]

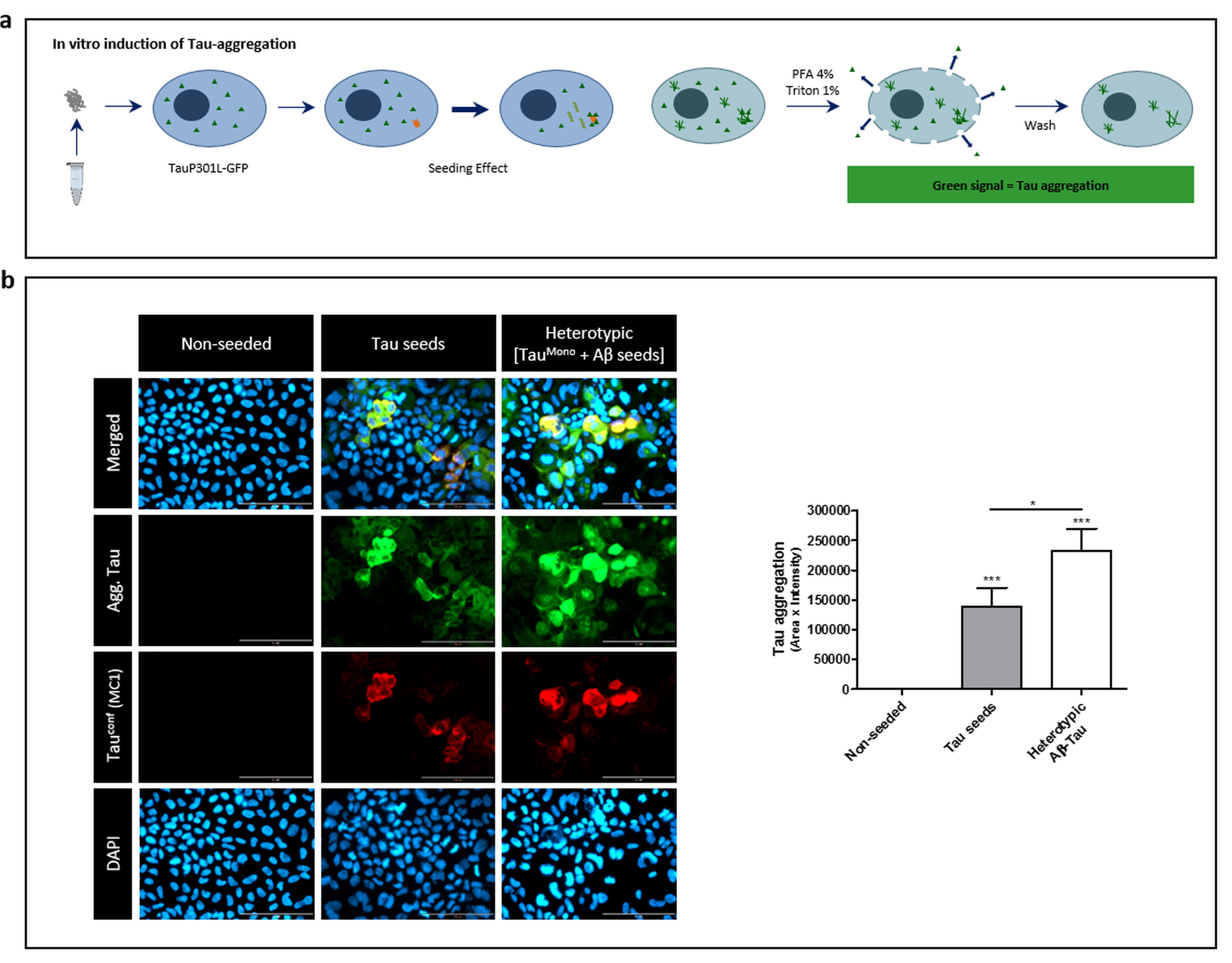

Supplement: Supplementary file 6 — Supplementary material 6 (TIFF 38713 kb) [file 401_2015_1525_MOESM6_ESM.tif]

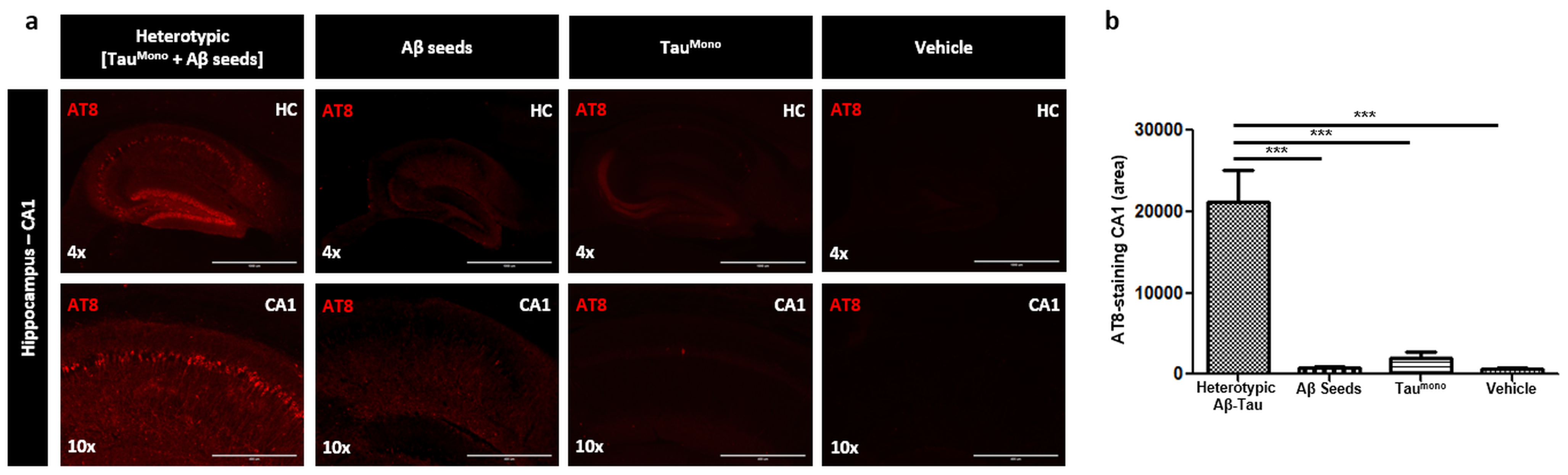

Supplement: Supplementary file 7 — Supplementary material 7 (TIFF 15001 kb) [file 401_2015_1525_MOESM7_ESM.tif]

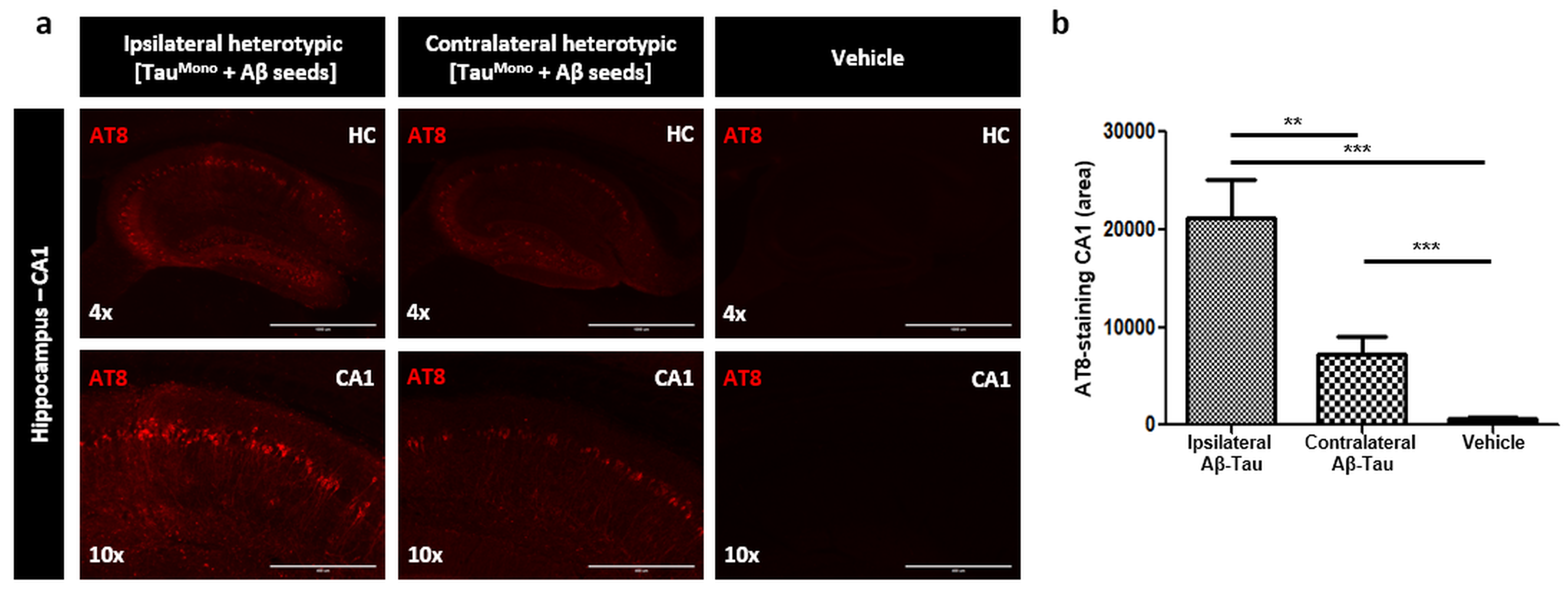

Supplement: Supplementary file 8 — Supplementary material 8 (TIFF 18700 kb) [file 401_2015_1525_MOESM8_ESM.tif]
