# Supplementary material for: Heterotypic seeding of Tau fibrillization by pre-aggregated Abeta provides potent seeds for prion-like seeding and propagation of Tau-pathology in vivo
Source: Acta Neuropathol. 2016 Jan 6;131:549–69. doi: 10.1007/s00401-015-1525-x (PMC4789256; doi:10.1007/s00401-015-1525-x)
Supplement: Supplementary file 9 — Supplementary material 9 (DOCX 25 kb) [file 401_2015_1525_MOESM9_ESM.docx]

**Supplemental data**

**Heterotypic seeding of Tau fibrillization by pre-aggregated Abeta provides potent seeds for prion-like seeding and propagation of Tau-pathology in vivo**

Bruno Vasconcelos^1†^, Ilie-Cosmin Stancu^1†^, Arjan Buist^2^, Matthew Bird^1^, Peng Wang^1^, Alexandre Vanoosthuyse^1^, Kristof Van Kolen^2^, An Verheyen^2^, Pascal Kienlen-Campard^1^, Jean-Noel Octave^1^, Peter Baatsen^3^, Diederik Moechars^2^, Ilse Dewachter^1*^

**Affiliations:**

^1^Alzheimer Dementia Group, Institute of Neuroscience, Catholic University of Louvain, 1200 Brussels, Belgium.

^2^Department of Neuroscience, Janssen Research and Development, A Division of Janssen Pharmaceutica NV, 2340 Beerse, Belgium.

^3^VIB11 vzw Center for the Biology of Disease, KU Leuven, 3000 Leuven, Belgium.

*Correspondence to: Ilse Dewachter ([ilse.dewachter@uclouvain.be](mailto:ilse.dewachter@uclouvain.be)), tel: (00 32) 2 764 93 36.

†The authors contributed equally to this work.

**Figures legends:**

**Fig. S1. Detailed characterization of the cellular Tau-aggregation assay**

**a. Detailed characterization of the cellular Tau-aggregation assay, demonstrating that Tau-seeding induces Tau-aggregation using biochemical and cytological analysis.**

**I.** Schematic overview of the Tau-seeded Tau-aggregation assay. Pre-aggregated Tau-seeds are added to QBI-293 cells overexpressing full-length TauP301L (GFP-tagged) using BioPORTER; inducing aggregation of TauP301L. **II.** Biochemical analysis reveals induction of Tau-phosphorylation (AT8) and a decreased mobility of Tau in SDS-Page following Tau-seeding (lower panels). Differential extraction of detergent insoluble Tau, reveals the presence of detergent insoluble Tau following Tau-seeding, not detected in non-seeded conditions (upper panel). **III.** Tau-aggregation is further demonstrated by cytological analysis. Stringent extraction with 1% TX-100 is used to eliminate soluble Tau-forms. Aggregated Tau is specifically detected following Tau-seeding while absent in non-seeded conditions. Tau-aggregates are stained with anti-phospho-Tau (S202/T205) antibody (AT8) - characteristic for pathological Tau in AD patient brains- while absent in non-seeded conditions (*scale bar* 100 µm).

**b. Detailed characterization of Tau-staining in the cellular Tau aggregation assay: Control experiments performed to demonstrate that strong colocalization of hyperphosphorylated Tau and aggregated Tau is not due to bleed-through.**

In view of the strong colocalization observed in the cellular Tau aggregation assay between aggregated total Tau and antibodies recognizing hyperphosphorylated Tau (AT8) and conformationally altered tau (MC1), appropriate controls are presented here to exclude bleed through. **I.** Following induction of Tau-aggregation by Tau-seeds, immunocytological analysis was performed following stringent detergent extraction to eliminate soluble forms of Tau as demonstrated (panel I). Cytological analysis was performed before AT8 staining (left panel) and after AT8 (right panel) coupled with goat anti-mouse Alexa 568 and analyzing the same fields with the GFP and Texas Red filters to exclude bleed through. This analysis demonstrated the absence of signal, in the absence of AT8 staining, using Texas Red filters and capture settings with similar and higher intensities than used for detection of AT8 stainings in our experimental setup. However, when the same field was imaged after staining with AT8, clear-cut AT8 staining was detected, using identical capture settings, demonstrating that this AT8 signal is not due to bleed-through. The obtained AT8 staining revealed strong colocalization with the pattern detected for total aggregated Tau (*scale bar* 100 µm). **II.** Similarly, staining using goat anti-mouse Alexa 647 coupled secondary antibody in combination with AT8 staining, in combination with GFP-filter and Cy5-filter to eliminate potential bleed through was performed. This revealed intensely labeled cells following AT8 staining, in a strikingly similar pattern as observed for aggregated Tau (*scale bar* 100 µm). **III.** Tau-GFP signal was extinguished using acid treatment, to demonstrate specificity of the staining. Panels represent stainings in ‘standard’ conditions and stainings obtained following ‘acid treatment’. This demonstrates that GFP-signal was extinguished in ‘acid conditions’, without eliminating the staining obtained with AT8. This demonstrates that the signal detected does not represent bleed through from the GFP-staining, but represents, Tau stained with AT8 antibody (*scale bar* 100 µm).

**Fig. S2. Pre-aggregated Tau and Aβ seeds, but not their monomeric forms induce Tau-aggregation in a well characterized in vitro Tau-aggregation assay.**

**a.** Schematic design of seed induced Tau-aggregation in QBI-293 cells expressing TauP301L-GFP. Pre-aggregated Tau seeds or Aβ seeds are added to the cells thereby bypassing the slow nucleation dependent lag phase. Aβ^Mono^ and Tau^Mono^ are also added to the cells to analyze if their sequence alone is capable of inducing Tau aggregation. Following stringent extraction using 1% TX-100 during PFA fixation, soluble forms of Tau are eliminated, the remaining GFP signal represents aggregated GFP-tagged TauP301L. **b.** Tau-seeds and Aβ-seeds induce Tau-aggregation in the in vitro Tau-aggregation assay, but not their monomeric counterparts (*scale bar* 400 µm). Quantitative analysis reveals significant induction of Tau aggregation only when Aβ-seeds and Tau seeds are used, no significant differences between their monomeric forms and non-seeded conditions are detected (n ≥ 20 fields per condition; one-way ANOVA followed by Tukey’s post hoc test; mean ± SEM are presented; **p* value <0.05, ***p* value <0.01, ****p* value <0.001). **c.** Higher magnification images of the different conditions are presented (*scale bar* 100 µm).

**Fig. S3. Pre-aggregated Aβ, but not pre-aggregated amylin, nor albumin, induce aggregation of monomeric Tau, following direct co-incubation.**

**a.** Schematic presentation of the different experimental conditions of incubation. Synthetic pre-aggregated Aβ peptides, synthetic pre-aggregated Tau-seeds, synthetic pre-aggregated amylin peptides, albumin and their respective buffer conditions are co-incubated with Tau^Mono^. **b.** Aggregation of Tau^Mono^ by the different seeds, was measured using ThioT assay at 1 hour, 1 day and 3 days post co-incubation. Quantitative analysis revealed significant induction of Tau-aggregation only by Aβ-seeds and Tau-seeds (n = 15; two-way ANOVA followed by Bonferroni post hoc test; mean ± SEM are presented; ****p* value <0.001).

**Fig. S4. Detailed characterization of immuno-electron microscopy analysis**

**a. Control stainings for immuno-electron microscopy demonstrate staining-specificity.**

To assure specific stainings using anti-Aβ and anti-Tau antibodies in immuno-EM, appropriate controls have been performed. These included immuno-EM staining of buffer only with anti-Aβ and anti-Tau antibody, demonstrating no staining under these conditions, excluding unspecific binding of the antibodies. Controls with no addition of primary antibodies revealed no staining, eliminating unspecific staining by the secondary antibodies used (results not shown). And staining of different concentrations of Tau^Mono^ revealed decreased staining of Tau^Mono^ with higher dilutions, further confirming specificity of the staining (*scale bar* 100 nm).

**b. Analysis of the Tau seeds and Aβ seeds used for the induction of monomeric Tau aggregation by immuno-electron microscopy.**

Highly concentrated sample of the pre-aggregated Tau and Aβ-seeds used for the induction of Tau^Mono^ aggregation were analyzed by immuno-electron microscopy with anti-Tau antibody (DAKO-Tau; 10 nm-gold labeled) and anti-Aβ antibody (WO2; 10 nm-gold labeled) before and after sonication. This demonstrated fibrillar Tau aggregates and fibrillar Aβ aggregates, which provided multiple pre-aggregated “seeds” following sonication (*scale bar* 100 nm).

**Fig. S5. Aβ-seeded Tau seeds Tau aggregation in a cellular Tau-aggregation assay**

**a.** Schematic presentation of the assay. Induction of Tau-aggregation was performed using Aβ-seeded Tau-seeds and Tau-seeds in TauP301L-GFP expressing cells. Analysis is performed by cytological analysis following stringent extraction to eliminate soluble forms of Tau. **b.** This analysis demonstrated the seeding potential for induction of Tau-aggregation by Aβ-seeded Tau seeds, reflected in induction of aggregated Tau, which is conformationally altered (MC1). Representative images of GFP-signal following stringent extraction with 1% TX-100, and immunofluorescent staining using antibody recognizing conformationally altered Tau (MC1) are presented, in control conditions and following seeding with Tau seeds and Aβ-seeded Tau seeds (*scale bar* 100 µm). Quantitative analysis reveals that Aβ-seeded Tau induces significantly higher Tau aggregation compared to Tau seeds (n ≥ 10 fields per condition; one-way ANOVA followed by Tukey’s post hoc test; mean ± SEM are presented; **p* value <0.05, ****p* value <0.001).

**Fig. S6. Induction of Tau-aggregation following seeding with Aβ-seeded Tau seeds in Tau transgenic mice in vivo**

**a.** Intracerebral injections of heterotypic Aβ-seeded Tau seeds, non-aggregated monomeric Tau, pre-aggregated Aβ-seeds and vehicle injections were performed in frontal cortex and hippocampus in Tau transgenic mice (4 months old). Tau-pathology was assessed using AT8 staining 3 months post-injection in frontal cortex and hippocampus. Representative hippocampal images of the different injection paradigms are shown demonstrating strong induction of Tau-pathology by Aβ-seeded Tau seeds compared to injection of non-seeded Tau^Mono^, pre-aggregated Aβ-seeds only or buffer injections (*scale bar* 1000 µm). This demonstrates the high efficiency of heterotypic Aβ-seeded Tau. Pre-aggregated Aβ-seeds induced only very limited Tau-pathology in Tau transgenic mice, comparable to limited Tau-pathology as demonstrated in previous publications, in line with low efficiency of heterotypic seeding. Higher magnifications are presented in the lower panels (*scale bar* 400 µm). No significant induction of motor phenotype, assessed by clasping and inverted grid hanging was observed following intracerebral injections in frontal cortex and hippocampus of heterotypic Aβ-seeded Tau seeds. **b.** Quantitative analysis of induced Tau-pathology revealed by AT8 staining in hippocampal CA1 region is presented. Induction of Tau-pathology following injection with heterotypic Aβ-seeded Tau was significantly higher compared to all other conditions analyzed (n = 6 per condition; one-way ANOVA followed by Tukey’s post hoc test; mean ± SEM are presented; ****p* value <0.001).

**Fig. S7. Aβ-seeded Tau propagate Tau pathology along functional connections in Tau transgenic mice in vivo.**

**a.** Representative AT8 stainings from the ipsi-lateral (injected) hippocampus and the contralateral (non-injected) hippocampus, following injection of heterotypic Aβ-seeded Tau, in comparison with AT8 staining of hippocampus of vehicle injected Tau transgenic mice 3 months post-injection, are shown (*scale bar* 1000 μm). Higher magnifications of hippocampal CA1 region of the ipsi-lateral side and contralateral side following seeding with heterotypic Aβ-seeded Tau, and hippocampal CA1 region of vehicle injected Tau mice are presented (*scale bar* 400 μm). This reveals clear-cut induction of Tau-pathology, at the contralateral side of heterotypic Aβ-seeded Tau injected mice, compared to vehicle injected brains, but less strong compared to the injected side, demonstrating propagation of Tau-pathology to remote but functionally connected brain regions. Tau-pathology stained with AT8 correlates strongly with silver stained Tau aggregates (results not shown). **b.** Quantitative analysis of AT8 staining in hippocampal CA1 region reveals significant induction of Tau-pathology in the non-injected hemisphere of heterotypic Aβ-seeded Tau injected brains (in frontal cortex and hippocampus), compared to age-matched vehicle injected mice, but less strong compared to the injected ipsilateral side, demonstrating propagation of Tau-pathology to remote but functionally connected brain regions (n = 6 per condition; one-way ANOVA followed by Tukey’s post hoc test; mean ± SEM are presented; ***p* value <0.01, ****p* value <0.001).
